# Functional and/or structural brain changes in response to resistance exercises and resistance training lead to cognitive improvements – a systematic review

**DOI:** 10.1186/s11556-019-0217-2

**Published:** 2019-07-10

**Authors:** Fabian Herold, Alexander Törpel, Lutz Schega, Notger G. Müller

**Affiliations:** 1 Research Group Neuroprotection, German Center for Neurodegenerative Diseases (DZNE), Leipziger Str. 44, 39120 Magdeburg, Germany; 2 Center for Behavioral Brain Sciences (CBBS), Brenneckestraße 6, 39118 Magdeburg, Germany; 3 Department of Neurology, Medical Faculty, Otto von Guericke University, Leipziger Str. 44, 39120 Magdeburg, Germany; 4 Institute III, Department of Sport Science, Otto von Guericke University Magdeburg, Zschokkestr. 32, 39104 Magdeburg, Germany

**Keywords:** Cognition, Neuroplasticity, Strength exercises, Strength training, Physical activity

## Abstract

**Background:**

During the aging process, physical capabilities (e.g., muscular strength) and cognitive functions (e.g., memory) gradually decrease. Regarding cognitive functions, substantial functional (e.g., compensatory brain activity) and structural changes (e.g., shrinking of the hippocampus) in the brain cause this decline. Notably, growing evidence points towards a relationship between cognition and measures of muscular strength and muscle mass. Based on this emerging evidence, resistance exercises and/or resistance training, which contributes to the preservation and augmentation of muscular strength and muscle mass, may trigger beneficial neurobiological processes and could be crucial for healthy aging that includes preservation of the brain and cognition. Compared with the multitude of studies that have investigated the influence of endurance exercises and/or endurance training on cognitive performance and brain structure, considerably less work has focused on the effects of resistance exercises and/or resistance training. While the available evidence regarding resistance exercise-induced changes in cognitive functions is pooled, the underlying neurobiological processes, such as functional and structural brain changes, have yet to be summarized. Hence, the purpose of this systematic review is to provide an overview of resistance exercise-induced functional and/or structural brain changes that are related to cognitive functions.

**Methods and results:**

A systematic literature search was conducted by two independent researchers across six electronic databases; 5957 records were returned, of which 18 were considered relevant and were analyzed.

**Short conclusion:**

Based on our analyses, resistance exercises and resistance training evoked substantial functional brain changes, especially in the frontal lobe, which were accompanied by improvements in executive functions. Furthermore, resistance training led to lower white matter atrophy and smaller white matter lesion volumes. However, based on the relatively small number of studies available, the findings should be interpreted cautiously. Hence, future studies are required to investigate the underlying neurobiological mechanisms and to verify whether the positive findings can be confirmed and transferred to other needy cohorts, such as older adults with dementia, sarcopenia and/or dynapenia.

## Background

### Aging, the brain, and cognition

Throughout the lifespan, the human organism undergoes considerable changes. As a consequence of aging, the structure and function of organic systems (i.e., brain) can be negatively affected, which in turn can converge in a decline of individual capabilities (e.g., cognition). In this regard, in recent years, evidence has shown that the hippocampus [1–4] and the grey matter in the frontal lobe [1–3, 5–12] are affected by age-related shrinking. In contrast, the grey matter volume of other brain structures such as the parietal and occipital cortices have been reported to change slightly with increasing age [1, 5, 8], whereas a severe decline in white matter volume of the prefrontal cortex (PFC) is most pronounced in the very oldest [1, 8, 9, 13, 14]. These age-related changes in brain structure [15, 16] are assumed to play major roles in the worsening of cognition functions, such as processing speed and memory [17–20]. In fact, in older adults, it was observed that a decrease in hippocampal volume is associated with worsening of memory performance [21–23]. Conversely, an increase in hippocampal volume after a yearlong aerobic training intervention was associated with memory improvements [24]. These findings suggest that the preservation of brain structures (e.g., hippocampus) is important to ensure the proper functioning of cognitive processes (e.g., memory). Similar to the relationship of brain structure and cognition, it is assumed that changes in brain function (e.g., brain activation during a cognitive task) contribute to changes in cognition [16, 25–27]. Such an intertwined relationship between brain activation and cognition is underpinned by the findings linking activation of the PFC to behavioral performance in executive function tasks [28–31], in visuomotor tasks [32], or in working memory tasks [33–35]. Currently, several hypotheses exist that aim to explain age-related alterations in brain activation and cognition [16, 25–27]. For instance, the HAROLD model predicts that there is hemispheric asymmetry reduction in older adults in the PFC during the execution of memory tasks [27, 36]. In the compensation-related utilization of the neural circuits hypothesis (CRUNCH), it is postulated that adults will recruit more brain regions (mainly the PFC) as the task load increases and that older adults need to recruit these brain regions at lower levels of cognitive load than younger adults (e.g., during working memory tasks) [26, 37–39]. In the Scaffolding Theory of Aging and Cognition (STAC), it is postulated that increased brain activity with age, especially in the PFC, is a compensatory mechanism caused by reorganization of the brain in response to the age-related decline in neural structures and neural functioning [16, 39, 40]. To date, none of these hypotheses satisfactorily explain the observed age-related changes in brain function [41], but all of these hypotheses emphasize the important role of the PFC in age-related functional brain changes. It is well recognized in the literature that physical exercises [28–30, 42, 43] and physical training [44–47] lead to positive changes in cognitive performance (e.g., executive functions) and brain activation patterns. Furthermore, the changes in brain activation patterns (i.e., shown by higher levels of oxygenated hemoglobin in brain regions) are associated with cognitive performance improvements [28–30, 47], which illustrate the important role of physical interventions in preserving cognition and brain health.

In summary, distinct cognitive functions (e.g., memory) are negatively affected, and substantial changes in brain structure (e.g., shrinkage of hippocampus) and brain function (e.g., compensatory brain activation; i.e., of PFC) occur as consequences of “normal” aging. Notably, regular engagement in physical exercise is a valuable strategy to counteract age-related decline in brain and cognition [48–52].

### Aging, muscular system, and cognition

There is solid evidence in the literature that muscle mass (sarcopenia) [53–57] and muscular strength (dynapenia) [53, 57–59], which constitute the ability to produce muscular force and power [60], decline gradually as a function of age. Notably, the age-related decrease in muscular strength was noticed to be more pronounced than the decrease in muscle mass [61–63]. Moreover, the decline in maximum muscular strength is more serious in the lower limbs than in upper limbs [62, 64–67]. In general, it was observed that the age-related loss in, for instance, maximum isokinetic hip/leg extensor strength is rather minimal until the fifth decade of life but accelerates considerably thereafter [58, 68–70]. Potential reasons for the pronounced decline in muscular strength are the reduction in cross-sectional area of the muscle fibers [64, 71] as well as the loss of muscle fibers and motor units [55, 56, 58, 61, 72, 73]. However, appropriate levels of muscular strength are needed for independent and healthy living. For instance, an appropriate level of muscular strength in the muscles of the lower limbs (e.g., hip and leg extensors) is required to ensure proper function for engaging in activities of daily living (e.g., balance and gait) [74, 75]. Hence, it is not surprising that a decline in isokinetic muscular strength in leg extensors is associated with reduced mobility [76–78] and increased risk of mortality [77, 79, 80].

However, there is growing evidence that an appropriate level of muscular strength is also linked to brain health and functioning (e.g., cognitive functions). In this regard, it has been reported in the literature that higher levels of isokinetic strength of the *M. quadriceps* *femoris* are linked to better performance in general cognitive abilities (operationalized by Mini-Mental State Examination [MMSE]) [81] and to better performance in executive functions [82, 83]. This link is further reinforced by the findings that higher leg power [84] and higher whole-body muscle strength [85] are associated with higher scores in standardized cognitive test batteries. Furthermore, higher handgrip strength is linked to higher scores in general cognitive abilities (e.g., operationalized by MMSE) [86, 87] and to higher scores in standardized cognitive test batteries [88–90]. Moreover, it was observed that gains in dynamic muscular strength (assessed by one repetition maximum in different resistance exercises) after 6 months of progressive resistance training mediate improvements in global cognitive performance (according to the Alzheimer’s Disease Assessment Scale–cognitive subscale) [91]. Similar to the previously mentioned finding, it was reported that changes in isokinetic knee extension and knee flexion torques after 3 months of progressive resistance training mediate improvements in executive functions [92]. Notably, a meta-analysis did not observe a correlation between muscle size and cognition [93] but reported that both muscle function (e.g., muscular strength) and muscle structure (e.g., muscle size) were linked to brain structure [93].

Taken together, during aging processes, a substantial decline in muscular strength, especially in lower limb muscles, occurs, and accumulating evidence suggests that lower muscular strengths are linked to poorer cognitive performance. Hence, resistance (strength) exercises (a single bout of resistance exercise, also referred to as acute exercise) and resistance (strength) training (more than one resistance exercise session, also referred to as chronic exercise; see also section ‘Data extraction’) seem to be promising activities to ensure the preservation of physical functioning and cognitive functions with aging.

### Resistance exercises, resistance training, brain, and cognition

One physical intervention strategy that is frequently recommended to counteract the age-related deterioration of both physical functioning and cognition is the continuous and regular execution of resistance exercises and/or resistance training [94–106]. There is solid evidence in the form of systematic reviews and meta-analyses indicating that resistance exercises and resistance training (for distinction, see section ‘Data extraction’) have substantial benefits for specific domains of cognitive functions (e.g., executive functions) [105, 107–111], but the underlying neurobiological mechanisms of resistance exercise-induced improvements in cognitive functions are not yet fully understood [107, 110].

**Fig. 1:**
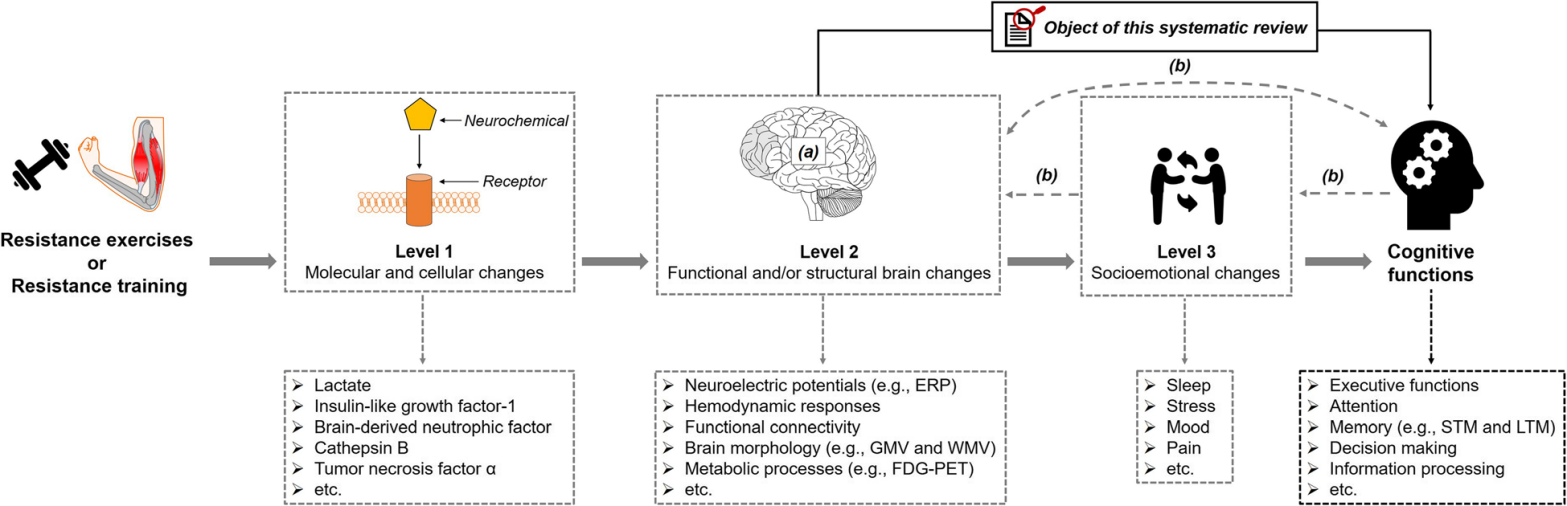
Schematic illustration of the objective of the present systematic review and the levels of analysis. ‘a’ indicates that the brain could be regarded as an outcome, a mediator or a predictor [122]. ‘b’ indicates several possibilities for how structural and functional brain changes, socioemotional changes, and cognitive changes are intertwined [112]. ERP: event-related potentials; FDG-PET: F-2-deoxy-D-glucose (FDG) positron-emissions tomography (PET); GMV: grey matter volume; LTM: long-term memory; STM: short-term memory; WMV: white matter volume

As shown in Fig. 1, cognitive improvements in response to resistance exercises and/or resistance training are based on changes on multiple levels of analysis [112, 113]. At the first level, molecular and cellular changes occur, which are summarized in the “neurotrophic hypothesis” [114–117]. The “neurotrophic hypothesis” claims that in response to physical exercises (e.g., resistance exercises), a pronounced release of distinct neurochemicals occurs (e.g., brain-derived neurotrophic factor [BDNF]) [114–117]. The pronounced release of specific neurochemicals triggers complex neurobiological processes evoking functional and/or structural brain changes that facilitate, at best, improvements in cognitive functions [24, 50, 114, 118–120]. With regard to the molecular and cellular levels, a systematic review summarized the evidence of resistance exercise and resistance training-induced changes in the release of several myokines (e.g., BDNF) and highlighted their positive effects on cognitive functions [121]. However, with respect to functional and structural brain changes and socioemotional changes (see Level 2 and Level 3 in Fig. 1), knowledge about resistance exercise and/or resistance training-induced changes is still relatively scarce, and the available literature has not yet been systematically pooled. In particular, the pooling of available evidence regarding functional and structural brain changes is needed because the brain may act as a mediator for the effect of resistance exercises and/or resistance training on cognition [112, 122]. Such a systematic pooling of available evidence is needed to provide evidence-based recommendations for individualized exercise prescriptions [123–125]. Because resistance exercises and/or resistance training is a promising strategy that could “hit many birds with one stone” (i.e., simultaneously counteracting different types of physical and brain-related health problems), the objective of this systematic review is to provide an overview of resistance exercise and/or resistance training-induced functional and/or structural brain changes that are related to changes in cognitive functions.

## Methods

### Search strategy and process

In accordance with the guidelines for systematic reviews [126], two independent researchers conducted a systematic literature search on the 25th of April 2019 across the following six electronic databases (applied specifications): PubMed (all fields), Scopus (title, abstract, keywords), Web of Science (title), PsycInfo (all text), SportDiscus (abstract), and the Cochrane Library (title, abstract, keywords; trials). The following terms were used as search strings:

- “strength exercise” OR “strength training” OR “resistance exercise” OR “resistance training” OR “weight exercise” OR “weight training” OR “weight lifting” OR “weight bearing” OR “elastic band” OR toning OR calisthenics OR “functional training”

AND

- mental OR neuropsychological OR brain OR cogniti* OR neurocogni* OR executive OR attention OR memory OR “response time” OR “reaction time” OR accuracy OR error OR inhibition OR visual OR spatial OR visuospatial OR processing OR recall OR learning OR language OR oddball OR “task switching” OR “problem solving” OR Flanker OR Stroop OR Sternberg OR “Trail Making” OR “Tower of London” OR “Tower of Hanoi” OR “Wisconsin Card Sorting” OR “Simon task”

AND

- cortex OR hemodynamic OR oxygenation OR “grey matter” OR “gray matter” OR “white matter” OR “brain volume” OR plasticity OR neuroelectric OR electrophysiological OR “P 300” OR “P 3” OR “event-related potentials” OR ERP OR Alpha OR Beta OR Gamma OR Theta OR NIR OR fNIRS OR “functional near-infrared spectroscopy” OR “near-infrared spectroscopy” OR “functional near-infrared spectroscopic” OR “optical imaging system” OR “optical topography” OR fMRI OR MRI OR “MR imaging” OR “magnetic resonance imaging” OR EEG OR electroencephalography OR electrocorticography OR MEG OR magnetoencephalography OR PET OR “positron emission tomography”

**Fig. 2:**
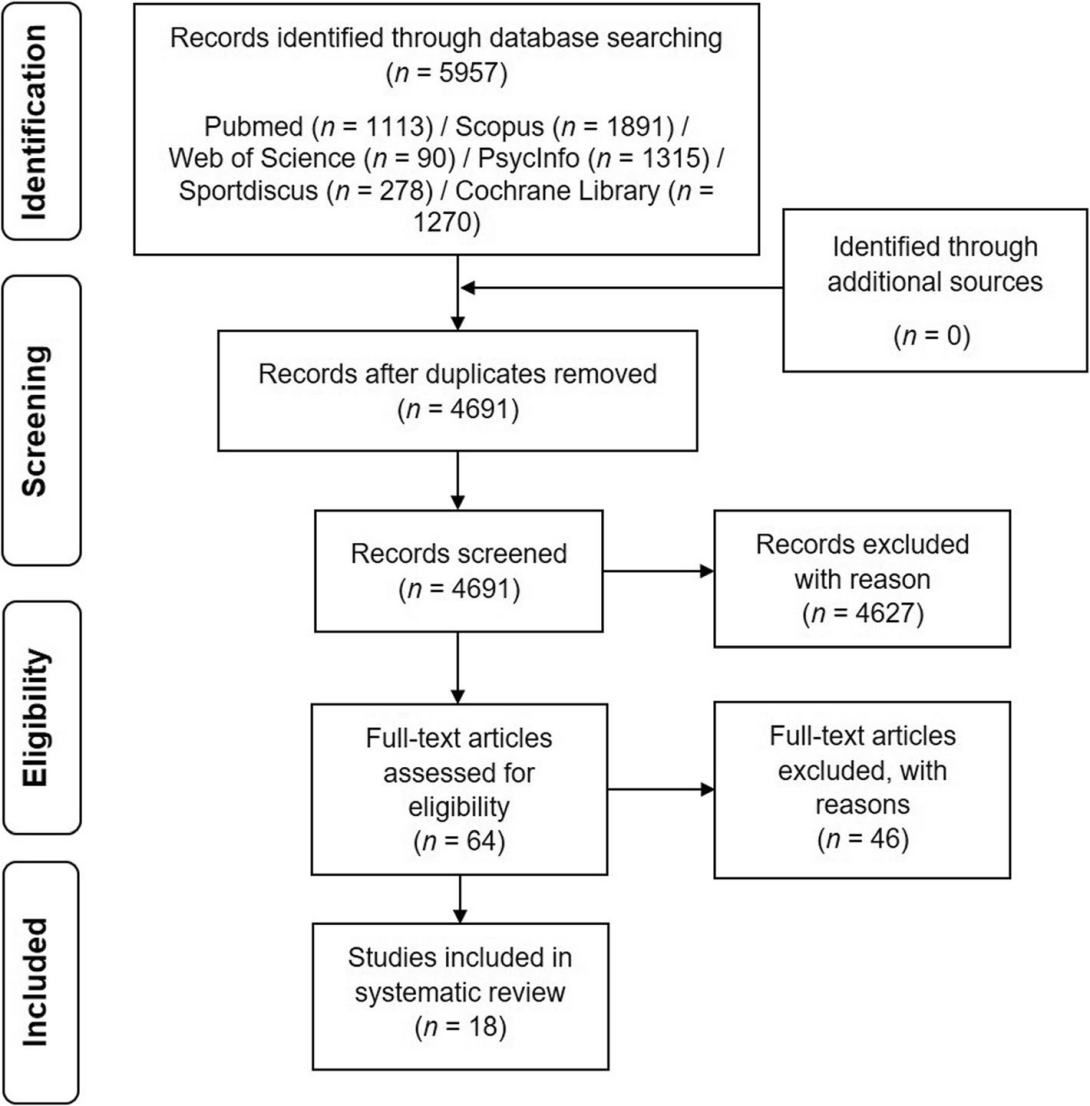
Flow chart with information about the search, screening, and selection processes that led to the identification of relevant articles included in this systematic review

Afterwards, the results of the systematic search were loaded into a citation manager (Citavi 6.3), which was used for further analyses and for removing duplicates (see Fig. 2).

### Inclusion and exclusion criteria

Screening for relevant studies was conducted using the established PICOS-principle [126, 127]. The acronym “PICOS” stands for participants (P), intervention (I), comparisons (C), outcomes (O), and study design (S) [126, 127]. The following inclusion and exclusion criteria were used: (P) we applied no restrictions and included all age groups regardless of pathologies; (I) only studies involving resistance exercises and/or resistance training were included; (C) in this systematic literature search, no specific restrictions were used; (O) studies considered relevant assessed functional brain changes and/or structural brain changes related to cognitive changes; (S) interventional or cross-sectional studies.

**Fig. 3:**
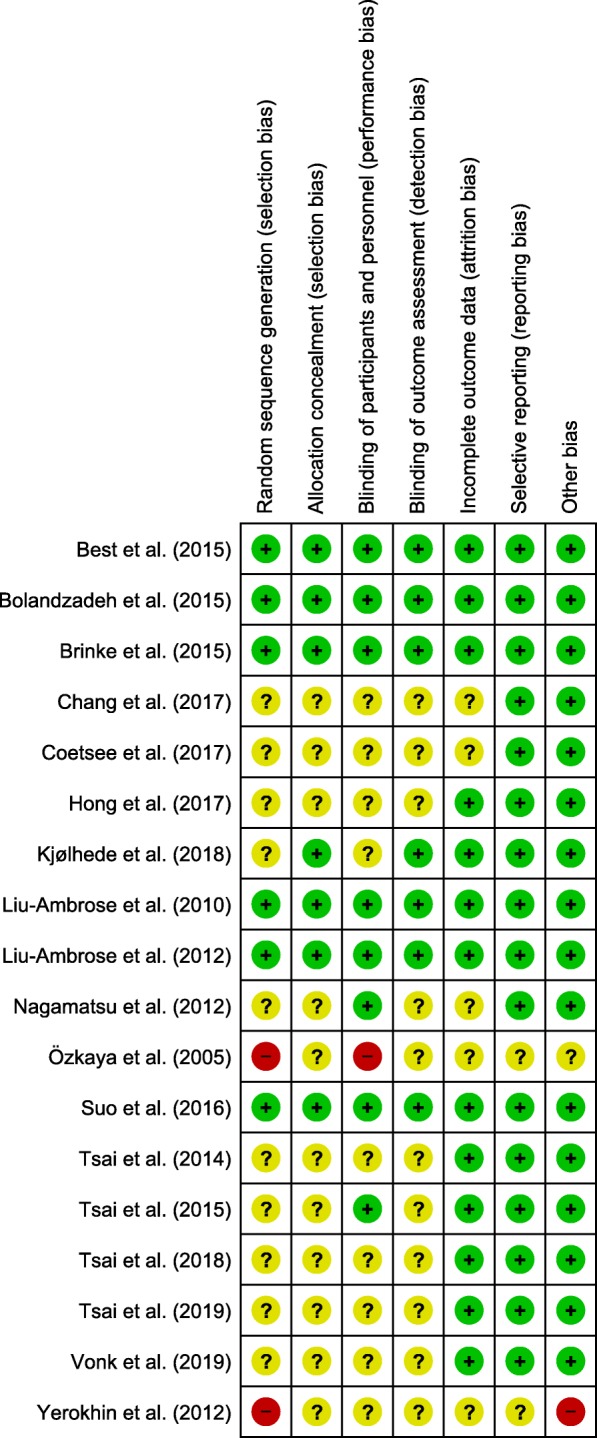
Analysis of the risk of bias in the included studies in accordance with the Cochrane Collaboration guidelines. This figure was created using Review Manager [172]. A “green plus” indicates a low risk of bias, a “yellow question mark” indicates an unclear risk of bias, and a “red minus” denotes a high risk of bias

As shown in Fig. 3, 46 studies were excluded after full text screening because they did not meet our inclusion criteria. Eight studies were excluded because they only assessed functional or structural brain changes but did not measure cognitive performance [128–135]. Vice versa, 38 studies were excluded because they solely measured changes in cognitive performance without quantifying functional or structural brain changes [81, 91, 136–171].

### Data extraction

We extracted information about the first author, year of publication, population characteristics including age, gender, cognitive status, exercise characteristics (e.g., muscle action, loading and volume, rest period between sets/between exercises, repetition velocity, frequency, resistance exercise selection), cognitive testing (e.g., tested cognitive domain, administration after exercise cessation), and functional and structural brain data. The extraction of information followed the recommendations of Hecksteden et al. [173].

Prior to presentation of the findings, it is necessary to clarify the different terms used in the field of exercise cognition. ‘Physical activity’ is defined as any muscle-induced bodily movements that increase energy expenditure from 1.0 to 1.5 MET [174, 175]. Hence, physical activity covers a wide range of acute and chronic physical activities (e.g., from housework to resistance exercises/resistance training). Specific forms of structured, planned, and regularly (chronically) conducted physical activities aiming to increase individual capabilities in a certain fitness domain are referred to as ‘training’ or ‘chronic (repetitive) exercises’ [174, 176–178]. Single sessions of physical activities (exercises) are referred to as ‘an acute (single) bout of physical activities’ or ‘physical exercises’ [174, 179, 180]. In this article, we use the term ‘resistance training’ when more than two exercise sessions were conducted. Consequently, a single session of resistance exercises is referred to as ‘a single (acute) bout of resistance exercises’ and/or ‘resistance exercises’. Furthermore, we use ‘exercise prescription’ as an umbrella term to denote exercise (e.g., load for an exercise) and training variables (e.g., frequency).

### Risk of bias assessment

Two evaluators independently performed the risk of bias assessment using the Cochrane Collaboration’s Risk of Bias tool [181]. The Cochrane Collaboration’s Risk of Bias tool evaluates the methodological quality of a study by rating the risk of bias in distinct criteria (see Figure 3) as being ‘low’, ‘high’, or ‘unclear’ [181]. Any discrepancies in the ratings of the risk of bias were resolved by a discussion among the two evaluators or/and the consultation of the third author of the review. The risk of bias assessment is summarized in Fig. 3.

## Results

### Risk of bias

As shown in Fig. 3, the results regarding the judgment of risk of bias are heterogeneous. In the domains of sequence generation, allocation concealment, blinding of participants and personnel, and blinding of outcome assessment, the majority of studies were rated as low risk of bias or unclear risk of bias. The reviewed studies were judged as having an unclear risk of bias in those domains because procedures were not described in sufficient detail (e.g., method of random sequence generation). In the domains of incomplete outcome data, selective reporting, and other bias, most studies were judged as having a low risk of bias.

### Participants’ characteristics and study design

In the reviewed studies, the effect of resistance exercises and/or resistance training on cognition and the brain was investigated in different cohorts, including healthy young adults [43, 182, 183], healthy older adults [44, 45, 184–188], older adults with mild cognitive impairment [188–191], older adults in an early stage of dementia [192], and individuals with multiple sclerosis [193]. Detailed information about participant characteristics (e.g., age, height, body mass) is provided in Table 1.

**Table 1:** Overview of the population characteristics and resistance exercises and/or resistance training characteristics of the reviewed studies

First author [ref.]	Study design and sample characteristics	Resistance exercise characteristics
	(1) Design / Comparison groups (2) Participants characteristics (2.1) Number of participants (N) (N female / N male), [included in fMRI or EEG], gender / mean age in years ± SD (2.2) Mean height in cm ± SD / mean body mass in kg ± SD / BMI ± SD in kg/m^2^ (3) Cognitive status / disability status	(1) Muscle action (2) Load, number of sets, and number of repetitions (3) Inter-set rest periods and inter-exercise rest periods (4) Repetition velocity (5) Resistance exercise selection (6) Duration of an exercise session (7) Training frequency (8) Training density (9) Training duration (10) Training setting
Functional near-infrared spectroscopy		
Chang et al. [43]	(1) IS (RCT, between-group design) / CON (n), HIRE, MIC, HIA (2) Healthy young adults (2.1) - CON: *N* = 9 (9 f / 0 m) / 21.8 ± 1.4 - HIRE: *N* = 9 (9 f / 0 m) / 21.1 ± 1.6 - MIC: *N* = 9 (9 f / 0 m) / 20.4 ± 1.5 - HIA: *N* = 9 (9 f / 0 m) / 22.1 ± 1.4 (2.2) - CON: 160.8 ± 4.1 / 52.2 ± 6.2 / 20.3 ± 3.1 - HIRE: 162.1 ± 5.0 / 56.3 ± 5.0 / 21.4 ± 1.8 - MIC: 162.9 ± 5.5 / 56.4 ± 5.8 / 21.2 ± 1.3 - HIA: 166.0 ± 5.3 / 59.6 ± 5.7 / 21.6 ± 2.1 (3) N.A.	(1) Dynamic (2) 3 sets with 8 to 10 repetitions per exercise at 80% of 1RM (3) Work to rest ratio of 1:2 (4) N.A. (5) Machines and free weights (e.g., leg extension, leg curl, lat pull-down, seated row, squat, bench press, and arm curl) (6) Ca. 40 min (10 min warm-up, 30 min exercising) (7) One single session (8) N.A. (9) N.A. (10) Individual and supervised
Coetsee et al. [44]	(1) IS (RCT, between-group design) / CON (n), HIIT, MCT, RT (2) Healthy older adults (2.1) - CON: *N* = 19 (11 f / 8 m) / 62.5 ± 5.6 - HIIT: *N* = 13 (10 f / 3 m) / 64.5 ± 6.3 - MCT: *N* = 13 (10 f / 3 m) / 61.6 ± 5.8 - RT: *N* = 22 (15 f / 7 m) / 62.4 ± 5.1 (2.2) - CON: 168.7 ± 7.9 / 76.8 ± 13.7 / 26.9 ± 3.7 - HIIT: 166.0 ± 8.9 / 73.8 ± 13.7 / 26.6 ± 4.0 - MCT: 163.5 ± 8.6 / 71.0 ± 14.4 / 26.5 ± 4.2 - RT: 167.8 ± 7.8 / 73.3 ± 15.5 / 25.8 ± 4.0 (3) MOCA score - CON: 28.2 ± 1.6 - HIIT: 27.9 ± 1.5 - MCT: 27.6 ± 1.3 - RT: 27.5 ± 1.3	(1) Dynamic (2) 3 sets with 10 repetitions per exercise at 50, 75, and 100% of 10RM (first 8 weeks) / at 75, 85, and 100% of 10RM (second 8 weeks) (3) N.A. (4) N.A. (5) Machines and free weights (e.g., upper and lower body resistance exercises) (6) Ca. 30 min (+ warm-up and cool-down) (7) 3 days/week (8) N.A. (9) 16 weeks (10) Group-based and supervised
Electroencephalography		
Hong et al. [188]	(1) IS (RCT, between-group design) / CON (n), RT (2) Healthy older adults / older adults with MCI (2.1) - HOA CON: *N* = 13 (6 f / 7 m) / 73.5 ± 5.6 (f); 73.0 ± 4.8 (m) - HOA RT: *N* = 12 (10 f / 2 m) / 75.8 ± 4.5 (f); 76.5 ± 6.4 (m) - MCI CON: *N* = 12 (9 f / 3 m) / 75.1 ± 4.5 (f); 78.3 ± 5.5 (m) - MCI RT: *N* = 10 (7 f / 3 m) / 75.1 ± 4.5 (f); 78.3 ± 5.5 (m) (2.2) - HOA CON: N.A. / 49.7 ± 4.5 (f); 63.4 ± 10.7 (m) / N.A. - HOA RT: N.A. / 57.3 ± 8.4 (f); 68.9 ± 4.7 / N.A. - MCI CON: N.A. / 56.3 ± 5.4 (f); 57.2 ± 7.6 (m) / N.A. - MCI RT: N.A. / 54.1 ± 7.6 (f); 65.0 ± 3.3 / N.A. (3) MOCA score - HOA CON: 26.0 ± 1.7 (f) / 26.3 ± 1.6 (m) - HOA RT: 26.4 ± 1.7 (f) / 25.0 ± 1.4 (m) - MCI CON: 18.8 ± 5.6 (f) / 21.3 ± 2.4 (m) - MCI RT: 20.0 ± 4.0 (f) / 22.3 ± 1.2 (m)	(1) Dynamic (2) 15 repetitions per exercise correspond to ca. 65% of 1RM (3) N.A. (4) N.A. (5) Elastic bands (6) Ca. 60 min (10 min warm-up, 40 min exercising, 10 min cool-down) (7) 2 days/week (8) N.A. (9) 12 weeks (10) N.A.
Özkaya et al. [194]	(1) IS (RCT, between-group design) / CON (n), AT, RT (2) Healthy older adults (2.1) - CON: *N* = 12 (N.A.) / 72.3 ± 2.1 - AT *N* = 12 (N.A.) / 70.9 ± 3.1 - RT: *N* = 12 (N.A.) / 75.8 ± 2.8 (2.2) - CON: N.A. / N.A. / 29.5 ± 1.3 - AT: N.A. / N.A. / 29.1 ± 1.4 - RT: N.A. / N.A. / 31.2 ± 2.9 (3) MMSE score - CON: 27.1 ± 0.6 - AT: 26.5 ± 0.6 - RT: 25.6 ± 0.7	(1) Dynamic (2) 1 set of 12 repetitions per exercise at 60% of 1RM (in the first week); 3 sets of 12 repetitions per exercise at 60% of 1RM (in the second week); increase in load of 5% every 2 weeks until participants lifted 80% of 1RM (3) N.A. (4) N.A. (5) Free weights (e.g., hip extension, knee flexion, seated lower-leg lift, chair squat, arm raise, biceps curl, and abdominal crunch) (6) N.A. (10 min warm-up, N.A., 10 min cool-down) (7) 3 days/week (8) N.A. (9) 9 weeks (10) Group-based and supervised
Tsai et al. [182]	(1) IS (RCT, between-group design) / CON (r), HIRE, MIRE (2) Healthy young adults (2.1) - CON: *N* = 20 (0 f / 20 m) / 23.2 ± 2.1 - MIRE: *N* = 20 (0 f / 20 m) / 23.2 ± 2.5 - HIRE: *N* = 20 (0 f / 20 m) / 22.4 ± 2.4 (2.2) - CON: N.A. / N.A. / 22.0 ± 2.6 - MIRE: N.A. / N.A. / 20.8 ± 1.5 - HIRE: N.A. / N.A. / 21.5 ± 1.8 (3) MMSE score - CON: 28.9 ± 0.9 - MIRE: 29.1 ± 1.0 - HIRE: 29.3 ± 1.0	(1) Dynamic (2) 2 sets of 10 repetitions per exercise at 50% of 1 RM in MIRT and at 80% of 1RM in HIRT (3) 90 s between sets / 2 min between exercises (4) “average speed” (5) Machines and free weights (e.g., bench presses, biceps curls, triceps extensions, leg presses, vertical butterflies, and leg extensions) (6) Ca. 40 min (10 min warm-up, 30 min exercising) (7) One single session (8) N.A. (9) N.A. (10) Individual and supervised
Tsai et al. [187]	(1) IS (RCT, between-group design) / CON (n), RT (2) Older adults (2.1) - CON: *N* = 24 (0 f / 24 m) / 72.0 ± 4.1 - RT: *N* = 24 (0 f / 24 m) / 70.8 ± 3.4 (2.2) - CON: N.A. / N.A. / 24.6 ± 3.6 - RT: N.A. / N.A. / 26.0 ± 2.5 (3) MMSE score - CON: 28.2 ± 1.0 - RT: 28.0 ± 1.2	(1) Dynamic (2) 3 sets of 10 repetitions per exercise at 75 to 80% of 1RM (3) 90 s between sets / 3 min between exercises (4) “average speed” (5) Machines and free weights (e.g., biceps curls, leg presses, triceps extensions, hamstring curls, latissimus dorsi pull-downs, calf raises, seated rowing) (6) Ca. 60 min (10 min warm-up, 40 min exercising, 10 min cool-down) (7) 3 days/week (8) N.A. (9) 48 weeks (10) Group-based and supervised
Tsai et al. [195]	(1) IS (RCT, between-group design) / CON (r), AE, RE (2) Older adults with amnestic MCI (2.1) - CON: *N* = 20 (12 f / 8 m) / 64.5 ± 7.0 - AE: *N* = 25 (14 f / 11 m) / 65.5 ± 7.5 - RE: *N* = 21 (12 f / 9 m) / 66.1 ± 6.6 (2.2) - CON: 159.7 ± 8.81 / 61.4 ± 13.0 / 23.8 ± 3.1 - AE: 160.6 ± 7.85 / 62.1 ± 13.7 / 23.8 ± 3.2 - RE: 159.9 ± 8.51 / 62.1 ± 12.1 / 24.5 ± 3.2 (3) MMSE score - CON: 27.00 ± 1.59 - AE: 26.96 ± 1.21 - RE: 26.76 ± 1.38	(1) Dynamic (2) 2 sets of 10 repetitions per exercise at 75% of 1RM (3) 90 s between sets / 2 min between exercises (4) “average speed” (5) Machines and free weights (e.g., biceps curls, triceps extensions, bench presses, leg presses, leg extensions, and vertical butterflies) (6) Ca. 40 min (5 min warm-up, 30 min exercising, 5 min cool-down) (7) One single session (8) N.A. (9) N.A. (10) Individual and supervised
Tsai et al. [191]	(1) IS (RCT, between-group design) / BAST, AT, RT (2) Older adults with amnestic MCI (2.1) - CON: *N* = 18 (13 f / 5 m) / 65.2 ± 7.0 - AT: *N* = 19 (14 f / 5 m) / 66.0 ± 7.7 - RT: *N* = 18 (11 f / 7 m) / 65.4 ± 6.8 (2.2) - CON: N.A. / N.A. / 23.4 ± 2.8 - AT: N.A. / N.A. / 23.5 ± 3.3 - RT: N.A. / N.A. / 24.4 ± 3.1 (3) MMSE score - CON: 27.00 ± 1.65 - AT: 27.16 ± 1.26 - RT: 26.56 ± 1.34	(1) Dynamic (2) 3 sets of 10 repetitions at 60 to 70% of 1RM in the first 2 weeks and at 75% of 1RM in the remaining weeks (3) 90 s between sets / 2 min between exercises (4) N.A. (5) Machines and free weights (e.g., biceps curls, vertical butterflies, leg press, seated rowing, hamstring curls, and calf raises) (6) Ca. 40 min (5 min warm-up, 30 min exercising, 5 min cool-down) (7) 3 days/week (8) N.A. (9) 16 weeks (10) Group-based and supervised
Vonk et al. [183]	(1) IS (RCT, within-subject design) / RE, LM (2) Healthy younger adults (2.1) *N* = 20 (11 f / 9 m) / 23.0 ± 2.0 (2.2) N.A. (3) N.A.	(1) Dynamic (2) 2 sets of 10 repetitions at 70% of 10RM (3) 60 s between sets / 90 min between exercises (4) N.A. (5) Machines and free weights (e.g., leg press, pull-down, hamstring curls, vertical chest press, bilateral bicep curl, bilateral triceps extension) (6) Ca. 30 min (5 min warm-up, ca. 25 min exercising) (7) Two separate sessions (RE and LM) (8) N.A. (9) N.A. (10) Individual and supervised
Yerokhin et al. [192]	(1) IS (no RCT, between-group design) / RT (2) Healthy older adults (2.1) - RT: *N* = 9 [5] (1 f / 8 m) / 62.8 ± 7.2 (2.2) - RT: N.A. / N.A. / N.A. Individuals with early dementia (2.1) - RT: *N* = 13 [9] (0 f /13 m) / 79.3 ± 11.0 (2.2) - RT: N.A. / N.A. / N.A. (3) MMSE score - N.A. in both groups	(1) Dynamic (2) N.A. (detailed information can be found in Seguin et al., [196]) (3) N.A. (detailed information can be found in Seguin et al., [196]) (4) N.A. (detailed information can be found in Seguin et al., [196]) (5) Small free weights and body weight (e.g., different exercise such as squat, toe stands, [detailed information could be found in Seguin et al., [196]) (6) Ca. 45 min (7) 3 to 5 days/week (8) N.A. (9) 10 weeks (10) Supervised (older adults with early dementia) / individual and home-based (HC)
Functional and structural magnetic resonance imaging		
Best et al. [184]	(1) IS (RCT, between-group design) / BAT, 1x RT, 2x RT (2) Older adults (2.1) - BAT: *N* = 49 [25/18/8] (49 f / 0 m) / 70.0 ± 3.3 - 1x RT: *N* = 54 [32/29/10] (54 f / 0 m) / 69.5 ± 2.7 - 2x RT: *N* = 52 [26/21/9] (52 f / 0 m) / 69.4 ± 3.0 (2.2) - BAT: 161.0 ± 6.9 / 67.0 ± 11.5 / N.A. - 1x RT: 160.9 ± 7.0 / 69.2 ± 16.2 / N.A. - 2x RT: 162.8 ± 6.5 / 72.1 ± 16.8 / N.A. (3) MMSE score - BAT: 28.8 ± 1.2 - 1x RT: 28.5 ± 1.3 - 2x RT: 28.6 ± 1.5	(1) Dynamic (2) 2 sets of 6 to 8 repetitions of 7RM per exercise (progressively increased) (3) N.A. (4) N.A. (5) Exercises with pneumatic resistance machines (e.g., biceps curls, triceps extensions, seated rows, latissimus dorsi pull-downs, leg presses, hamstring curls, and calf raises) and free weights (e.g., mini-squats, mini-lunges, and lunge walks) (6) Ca. 60 min (10 min warm-up, 40 min exercising, 10 min cool-down) (7) 1 day/week (in 1x RT) or 2 days/week (in 2x RT) (8) One week-in-between (in 1x RT) / N.A. (in 2x RT) (9) 52 weeks (10) Group-based and supervised
Brinke et al. [197]	(1) IS (RCT, between-group-design) / BAT, AT, RT (2) Older adults with probable MCI (2.1) - BAT: *N* = 28 [13/11] (28 f / 0 m) / 75.5 ± 3.9 - AT: *N* = 30 [14/10] (30 f / 0 m) / 76.1 ± 3.4 - RT: *N* = 28 [12/8] (30 f / 0 m) / 73.8 ± 3.8 (2.2) - BAT: 157.5 ± 8.1 / 64.8 ± 13.8 / N.A. - AT: 158.8 ± 5.8 / 61.7 ± 6.8 / N.A. - RT: 161.6 ± 8.1 / 63.3 ± 7.5 / N.A. (3) MMSE score - BAT: 27.17 ± 1.85 - AT: 27.54 ± 1.51 - RT: 26.67 ± 2.64	(1) Dynamic (2) 2 sets of 6 to 8 repetitions of 7RM per exercise (progressively increased) (3) N.A. (4) N.A. (5) Exercises with pneumatic resistance machines (e.g., biceps curls, triceps extensions, seated rows, latissimus dorsi pull-downs, leg presses, hamstring curls, and calf raises) and free weights (e.g., mini-squats, mini-lunges, and lunge walks) (6) Ca. 60 min (10 min warm-up, 40 min exercising, 10 min cool-down) (7) 2 days/week (8) N.A. (9) 26 weeks (10) Group-based and supervised
Bolandzadeh et al. [185]	(1) IS (RCT, between-group design) / BAT, 1x RT, 2x RT (2) Older adults (2.1) - BAT: *N* = 15 [11] (15 f / 0 m) / 69.3 ± 2.8 - 1x RT: *N* = 22 [18] (22 f / 0 m) / 69.6 ± 2.6 - 2x RT: *N* = 17 [13] (17 f / 0 m) / 69.2 ± 3.1 (2.2) - BAT: 162.9 ± 5.8 / 69.5 ± 9.4 / N.A. - 1x RT: 160.7 ± 6.4 / 68.2 ± 14.6 / N.A. - 2x RT: 161.3 ± 7.4 / 68.1 ± 12.5 / N.A. (3) MMSE (MOCA) score - BAT: 28.7 (24.4) ± 1.3 (3.5) - 1x RT: 28.9 (25.8) ± 1.0 (2.9) - 2x RT: 28.8 (25.6) ± 1.8 (2.9)	(1) Dynamic (2) 2 sets of 6 to 8 repetitions of 7RM per exercise (progressively increased) (3) N.A. (4) N.A. (5) Exercises with pneumatic resistance machines (e.g., biceps curls, triceps extensions, seated rows, latissimus dorsi pull-downs, leg presses, hamstring curls, and calf raises) and free weights (e.g., mini-squats, mini-lunges, and lunge walks) (6) Ca. 60 min (10 min warm-up, 40 min exercising, 10 min cool-down) (7) 1 day/week (in 1x RT) or 2 days/week (in 2x RT) (8) One week-in-between (in 1x RT) / N.A. (in 2x RT) (9) 52 weeks (10) Group-based and supervised
Kjølhede et al. [193]	(1) IS (RCT, cross-over design) / WL, RT (2) Adults with multiple sclerosis (2.1) - WL: *N* = 17 [12] (N.A.) - RT: *N* = 18 [17] (N.A.) - mean of both groups: 43.2 ± 8.1 (2.2) - mean of both groups: 171.0 ± 8.0 / 75.0 ± 13.0 / N.A. (3) EDSS score - WL: 2.9 ± 0.2 - RT: 2.9 ± 0.2	(1) Dynamic (2) Progressively increased with adjustment in sets, repetitions, load [detailed information can be found in Kjølhede et al. [198] (3) 2 to 3 min [detailed information can be found in Kjølhede et al. [198] (4) N.A. (5) Exercises with resistance machines (e.g., horizontal leg press, hip flexion, leg extension, prone hamstring curl, cable pull-down and cable triceps extension) (6) N.A. (7) 2 days/ week (8) N.A. (9) 24 weeks (10) Group-based and supervised
Liu-Ambrose et al. [186]	(1) IS (RCT, between-group design) / BAT, 1x RT, 2x RT (2) Older adults (2.1) - BAT: *N* = 49 [20/18] (49 f / 0 m) / 70.0 ± 3.3 - 1x RT: *N* = 54 [28] (54 f / 0 m) / 69.5 ± 2.7 - 2x RT: *N* = 52 [18] (52 f / 0 m) / 69.4 ± 3.0 (2.2) - BAT: 161.0 ± 6.9 / 67.0 ± 11.5 / N.A. - 1x RT: 160.9 ± 7.0 / 69.2 ± 16.2 / N.A. - 2x RT: 162.8 ± 6.5 / 72.1 ± 16.8 / N.A. (3) MMSE score - BAT: 28.8 ± 1.2 - 1x RT: 28.5 ± 1.3 - 2x RT: 28.6 ± 1.5	(1) Dynamic (2) 2 sets of 6 to 8 repetitions of 7RM per exercise (progressively increased) (3) N.A. (4) N.A. (5) Exercises with pneumatic resistance machines (e.g., biceps curls, triceps extensions, seated rows, latissimus dorsi pull-downs, leg presses, hamstring curls, and calf raises) and free weights (e.g., mini-squats, mini-lunges, and lunge walks) (6) Ca. 60 min (10 min warm-up, 40 min exercising, 10 min cool-down) (7) 1 day/week (in 1x RT) or 2 days/week (8) One week-in-between (in 1x RT) / N.A. (in 2x RT) (9) 52 weeks (10) Group-based and supervised
Liu-Ambrose et al. [45]	(1) IS (RCT, between-group design) / BAT, 1x RT, 2x RT (2) Older adults (2.1) - BAT: *N* = 17 [17] (17 f / 0 m) / 69.2 ± 3.2 - 1x RT: *N* = 20 [20] (20 f / 0 m) / 69.7 ± 2.8 - 2x RT: *N* = 15 [15] (15 f / 0 m) / 68.9 ± 3.2 (2.2) - BAT: 162.4 ± 5.9 / 67.3 ± 9.5 / N.A. - 1x RT: 161.7 ± 7.5 / 70.7 ± 13.8 / N.A. - 2x RT: 162.7 ± 6.6 / 68.7 ± 10.9 / N.A. (3) MMSE score - BAT: 29.1 ± 1.1 - 1x RT: 28.6 ± 1.2 - 2x RT: 29.1 ± 0.85	(1) Dynamic (2) 2 sets of 6 to 8 repetitions of 7RM per exercise (progressively increased) (3) N.A. (4) N.A. (5) Exercises with pneumatic resistance machines (e.g., biceps curls, triceps extensions, seated rows, latissimus dorsi pull-downs, leg presses, hamstring curls, and calf raises) and free weights (e.g., mini-squats, mini-lunges, and lunge walks) (6) Ca. 60 min (10 min warm-up, 40 min exercising, 10 min cool-down) (7) 1 day/week (in 1x RT) or 2 days/week (in 2x RT) (8) One week-in-between (in 1x RT) / N.A. (in 2x RT) (9) 52 weeks (10) Group-based and supervised
Nagamatsu et al. [189]	(1) IS (RCT, between-group design) / BAT, 2x AT, 2x RT (2) Older adults with probable mild cognitive impairment and subjective memory complaints (2.1) - BAT: *N* = 28 [8] (28 f / 0 m) / 75.1 ± 3.6 - AT: *N* = 30 [7] (30 f / 0 m) / 75.6 ± 3.6 - RT: *N* = 28 [7] (28 f / 0 m) / 73.9 ± 3.5 (2.2) - BAT: 158.2 ± 7.3 / 66.4 ± 14.0 / N.A. - AT: 159.2 ± 5.9 / 64.8 ± 12.8 / N.A. - RT: 158.7 ± 7.0 / 65.2 ± 10.7 / N.A. (3) MMSE (MOCA) score - BAT: 27.1 (22.5) ± 1.7 (2.8) - AT: 27.4 (22.2) ± 1.5 (2.8) - RT: 27.0 (21.4) ± 1.8 (1.3)	(1) Dynamic (2) 2 sets of 6 to 8 repetitions of 7RM per exercise (progressively increased) (3) N.A. (4) N.A. (5) Exercises with pneumatic resistance machines (e.g., biceps curls, triceps extensions, seated rows, latissimus dorsi pull-downs, leg presses, hamstring curls, and calf raises) and free weights (e.g., mini-squats, mini-lunges, and lunge walks) (6) Ca. 60 min (10 min warm-up, 40 min exercising, 10 min cool-down) (7) 2 days/week (8) N.A. (9) 52 weeks (10) Group-based and supervised
Suo et al. [190]	(1) IS (RCT, between-group design) / SHAM, RE + SHAM, RE + CCT, CCT + SHAM (2) Older adults with dementia prodrome mild cognitive impairment (2.1) - ALL: *N* = 100 (68 f / 32 m) / 70.1 ± 6.7 (55–87) - SHAM: *N* = 27 [22] (N.A.) - RE + SHAM: *N* = 22 [19] (N.A.) - RE + CCT: *N* = 27 [22] (N.A.) - CCT + SHAM: *N* = 24 [20] (N.A.) (2.2) - N.A. (3) MMSE score - ALL: 24–28 (29 was acceptable only if error noted in memory registration)	(1) Dynamic (2) 5 to 6 exercises with 3 sets of 8 repetitions per exercise at 80 to 92% of 1RM (3) N.A. (4) N.A. (5) Exercises with pneumatic resistance machines (e.g., chest press, leg press, seated row, standing hip abduction, knee extension, hip flexion, hip extension, calf raise) and free weights (e.g., lateral raise, biceps curls) (6) Ca. 90 min (7) 2 days/week (8) N.A. (9) 26 weeks (10) Group-based and supervised

Please note that the sham treatments in Suo et al. [190] were conducted as follows: (i) the cognitive training group (CCT + SHAM) included physical exercises that did not significantly increase heart rate or improve aerobic capacity balance or strength performance (e.g., stretching, toning, and seated calisthenics), and (ii) the resistances exercise group (RE + SHAM) included a computerized, active cognitive control training

*AE* Aerobic exercises, *AT* Aerobic training, *BAT* Balance and toning exercise, *BAST* Balance and stretching training, *BMI* Body mass index, *cm* Centimeters, *CON (n)* Non-exercising control group, *CON (r)* Control group read magazines, *EDSS* Expanded disability status scale, *f* Female, *HIA* High-intensity aerobic exercise, *HIIT* High-intensity aerobic interval training, *HIRE* High-intensity resistance exercises, *HIRT* High-intensity resistance training, *HOA* Healthy older adults, *kg* Kilogram, *LM* Loadless movement group, *MCI* Mild cognitive impairments, *MIC* Moderate-intensity exercise combining resistance training and walking, *MCT* Moderate continuous aerobic training, *MIRE* Moderate-intensity resistance exercises, *m* Male, *min* Minute, *MMSE* Mini-mental state examination, *MOCA* Montreal cognitive assessment, *N* Number of participants, *N.A.* Not applicable, *RCT* Randomized controlled trials, *RM* Repetition maximum, *RE* Resistance exercises, *RT* Resistance training, *SD* Standard deviation, *WL* Wait list

Regarding the study design, almost all studies could be classified as interventional and as randomized controlled trials [43–45, 183–186, 188–190, 195, 197].

Additionally, three resistance exercise studies [43, 182, 183, 195] accounted for circadian variability as a possible moderating factor.

### Resistance exercise characteristics

In four studies investigating the acute effects of single resistance exercise sessions on cognitive performance and on functional neuroelectric or hemodynamic brain processes, the exercise sessions lasted approximately 30 min [183] or 40 min [43, 182, 195].

Studies on the effects of resistance training on cognition and functional and/or structural brain changes involved groups who trained 1 day [45, 184–186], 2 days [45, 184–186, 188–190, 193, 197], or 3 days per week [44, 187, 191]. Exercise sessions in the resistance training studies lasted 30 min [44], 40 min [191], 60 min [45, 184–189, 197] or 90 min [190]. The regimes were conducted for 9 weeks [194], 10 weeks [192], 12 weeks [188], 16 weeks [44, 191], 24 weeks [193], 26 weeks [190, 197], 48 weeks [187], or 52 weeks [45, 184–186, 189]. In most of the resistance training studies reviewed, the exercise sessions were conducted in supervised classes [44, 45, 184–187, 189–191, 193, 197]. Furthermore, in most of the reviewed studies, participants were asked to perform two or three sets during the exercise sessions with a minimum of six and a maximum of ten repetitions of upper and lower body exercises at a load ranging from 50 to 92% of 1RM (one repetition maximum) using free weights and/or machines (for a detailed overview, see Table 1).

## Main findings

### Functional brain changes and cognition

#### Hemodynamic functional brain changes and cognition

With regard to an acute bout of resistance exercises, in healthy young adults, a decrease in tissue oxygenation index in the left prefrontal cortex during the Stoop test and improved behavioral performance (i.e., faster reaction time and higher number of solved items in neutral condition) was observed after a single bout of high-intensity resistance exercise [43].

With regard to resistance training, after a 16-week intervention with healthy older adults, oxygenated hemoglobin and total hemoglobin were lowered in the left prefrontal cortex during the Stroop task (Stroop interference effect, posttest compared with pretest), while cognitive task performance (i.e., reaction time) was improved [44]. At the end of 52 weeks of resistance training, older adults who had conducted resistance exercises twice a week exhibited better performance in tasks of executive functions (i.e., Stroop test) than those who had performed balance and toning exercises [45]. Furthermore, in the same study, the hemodynamic response during the incongruent flanker condition was increased in the left anterior insula and the left lateral orbitofrontal cortex, whereas the hemodynamic response during the congruent flanker condition decreased in the same areas [45].

In older individuals with mild cognitive impairment (MCI), the right lingual and occipital-fusiform gyri and the right frontal pole exhibited increased activation during the associative memory test after a twice-weekly performed resistance training lasting for 52 weeks when compared with older individuals conducting balance and toning exercises in this time period [189]. Furthermore, in this study, a positive correlation between increased hemodynamic activity in the right lingual gyrus and improved associative memory performance was observed [189]. After 26 weeks of resistance training, decreased resting-state functional connectivity of the PC_FC_ with the left inferior temporal lobe and the anterior cingulate cortex and between the HIP_FC_ and the right inferior temporal lobe was observed in older adults with MCI [190]. In the same study, an increase in resting-state functional connectivity between the HIP_FC_ and the right middle frontal lobe was evident in older adults with MCI in the resistance training group [190].

#### Neuroelectric functional brain changes and cognition

With regard to an acute bout of resistance exercises, cognitive performance was improved in younger adults [182, 183] and older adults with MCI [195]. After exercising in younger adults, an increase in the P3 amplitude during a Go/No-Go task combined with the Eriksen Flanker paradigm was observed [182], and in older adults with MCI, the P3 amplitude across all electrode positions (except Pz) during the Eriksen Flanker task was larger posttest compared with pretest [195]. Furthermore, in younger adults, a time-dependent and condition-dependent increase in P3 amplitude (obtained during the Stroop task) was observed [183]. In incongruent trials, larger P3 amplitudes were observed 30 min and 40 min after exercise cessation, whereas in congruent trials, larger P3 amplitudes were observed 10 min and 40 min after exercise cessation [183]. However, in the same study, no statistically significant differences between the resistance exercise group and the loadless movement group were observed [183]. Additionally, larger P3 amplitudes were associated with lower serum cortisol levels after an acute bout of resistance exercise in younger adults [182].

With regard to resistance training, after 9 weeks of training (three times per week), the elderly participants showed a significant decrease in N1 latencies at the Fz and Cz positions during an auditory task, whereas the N1-P2, P2-N2 and N2-P3 amplitudes (at Fz) and the N1-P2 amplitude (at Cz) increased [194]. In comparison to both an aerobic training group and an inactive control group, the resistance training group showed  a greater absolute reduction in P2 and N2 latencies and larger absolute increase in N1-P2, P2-N2, and N2-P3 amplitudes [194]. Furthermore, after 10 weeks of resistance training in healthy older adults and in older adults at an early stage of dementia, a decrease in beta asymmetry, a decrease in N200 A asymmetry, and an increase in theta asymmetry was observed [192]. The decrease in N200 A asymmetry was significantly negatively correlated with improvements in the Fuld immediate recall score and the Fuld delayed recall score, while the increase in delta asymmetry was significantly positively correlated with a better Fuld delayed recall score [192]. After resistance training with elastic bands for 12 weeks, healthy older adults showed a decrease in relative theta power at P3 and P4, but their cognitive measures remained unchanged [188]. However, in the same study, exercising older adults with MCI exhibited significantly higher scores in the digit span backward test than their non-exercising counterparts [188]. Furthermore, from pre- to posttest, theta power at F3 increased and alpha power at T3 decreased in exercising older adults with MCI [188]. After 16 weeks of resistance training in older adults with amnestic MCI, larger P3 amplitudes during a task-switching paradigm were observed [191]. Furthermore, in the same study, decreased reaction times (i.e., in the non-switching condition and in the switching condition) and higher accuracy rates (i.e., in the pure condition, in the non-switching condition, and in the switching condition) were noticed in the resistance training group and the aerobic training group when the posttest was compared with the pretest [191]. Additionally, in the resistance training group, a positive correlation between changes in serum levels of insulin-like growth factor 1 (IGF-1) and P3 amplitudes (measured during switching condition) and a negative correlation between serum levels of tumor necrosis factor-alpha and accuracy rates in the switching condition were observed, which both barely failed to attain statistical significance [191]. In another study, 48 weeks of resistance training led to superior cognitive performance (i.e., reaction time) as well as to larger P3a and P3b amplitudes in an oddball task [187]. Moreover, serum IGF-1 concentrations increased and were correlated with faster reaction times and larger P3b amplitudes only in the resistance group [187].

### Structural brain changes and cognition

After resistance training performed once or twice weekly for 52 weeks, compared with older adults conducting balance and toning exercises, older adults in the resistance training groups exhibited (i) an increased performance in Stroop test [186], (ii) a reduction in whole brain volume [186], (iii) a lower volume of cortical white matter atrophy [184], and (iv) a lower degree of cortical white matter lesions [185]. In older female adults with probable MCI, resistance training over 26 weeks did not led to significant changes in hippocampal volume [197]. In another study, older adults with MCI resistance training performed twice a week for 26 weeks exhibited improved ADAS-Cog scores (global cognition assessed with Alzheimer’s Disease Assessment Scale) and increased the cortical thickness of grey matter in the posterior cingulate gyrus [190]. Moreover, the increase in grey matter thickness was negatively correlated with ADAS-Cog scores, indicating better cognitive performance [190]. In individuals with multiple sclerosis (MS), resistance training lasting 24 weeks led to an increase in cortical thickness in the anterior cingulate sulcus and gyrus, the temporal pole, the inferior temporal sulcus, and the orbital H-shaped sulcus [193]. The increased thickness in the temporal pole was significantly negatively correlated with lower scores on the Expanded Disability Status Scale (i.e., lower disability) [193]. More detailed information on the main findings is provided in Table 2.

**Table 2:** Overview of the characteristics of cognitive testing and the main outcomes of the reviewed studies

First author [ref.]	(1) Cognitive testing
	(2) Main findings (related to functional and/or structural brain changes in response to resistance exercises or resistance training)
Functional near-infrared spectroscopy	
Chang et al. [43]	(1) Executive functions (Stroop test) during fNIRS (conducted 15 min after exercise cessation)
	(2) Between group comparisons (postexercise, neutral condition):
	- ↓ TOI in lt. PFC during CT (HIR vs. CON (n) / MIC)
	- ↑ Solved items and ↓ response time during CT (HIR vs. CON (n))
	Between group comparisons (postexercise, incongruent condition):
	- ↓ TOI in lt. PFC (HIR vs. CON (n) / MIC)
	- ↓ TOI in rt. PFC (HIR vs. CON (n) / MIC / HIA)
	(ROI: lt. and. rt. PFC)
Coetsee et al. [44]	(1) Executive functions (Stroop test) during fNIRS
	(2) Posttest vs. pretest:
	- ↓ OxyHb in lt. PFC in RT during CT (Stroop interference effect)
	- ↓ THI in lt. PFC in RT and MCT during CT (Stroop interference effect)
	- ↓ Reaction time in RT during CT (naming and executive condition)
	(ROI: lt. and rt. PFC)
Electroencephalography	
Hong et al. [188]	(1) Cognitive test battery (Stroop test, COWAT, DFDB; Rey 15-Item Memory Test) and resting EEG
	(2) Posttest versus pretest:
	- ↓ Relative theta power (at F3) in MCI RT
	- ↑ Relative alpha power (at T3) in MCI RT
	- ↓ Relative theta power (at P3 and at P4) in HOA RT
	- DB scores were significantly higher in MCI RT than in MCI CON (at posttest)
Özkaya et al. [194]	(1) Auditory task during EEG
	(2) Posttest vs. pretest:
	- ↓ Latencies of N1 (at Fz) and N1 (at Cz) in RT and AT
	- ↑ Amplitudes of N1-P2, P2-N2 and N2-P3 (at Fz) and N1-P2 (at Cz) in RT
	Between group comparisons:
	- ↓ Absolute changes in latencies of P2 and N2 (at Fz and at Cz) in RT compared with AT and CON
	- ↑ Absolute changes in amplitudes of N1-P2, P2-N2, and N2-P3 (at Fz) and N1-P2 and N2-P3 (at Cz) in RT compared with AT and CON
Tsai et al. [182]	(1) Executive functions (Go/No-Go task combined with the Eriksen Flanker paradigm) during EEG measurements (CT was conducted after exercise cessation when the participant’s body temperature and HR had returned to within 10% of pre-exercise levels, which was on average approximately 5 min after acute resistance exercise cessation.)
	(2) Posttest vs. pretest:
	- ↑ P3 amplitude (i.e., at Fz, Cz, and Pz) in MIRT and HIRT during CT
	- ↓ Reaction time in MIRT and HIRT during CT (Go condition)
	- ↑ Accuracy in MIRT and HIRT during CT (incongruent No-Go condition)
	- ↑ Serum GH and serum IGF-1 in MIRE and HIRE (prior to cognitive testing at pretest vs. prior to cognitive testing at posttest)
	- ↓ Serum cortisol in MIRE and HIRE (prior to cognitive testing at pretest vs. prior cognitive testing at posttest)
	- ↓ Serum GH and serum IGF-1 in HIRE (prior to cognitive testing at posttest vs. after cognitive testing at posttest)
	- ↑ Serum GH in MIRE and HIRE, serum IGF in MIRE (prior to cognitive testing at pretest vs. after cognitive testing at posttest)
	- ↓ Serum cortisol in MIRE (prior to cognitive testing at pretest vs. after cognitive testing at posttest)
	- Lower serum cortisol levels were associated with higher P3 amplitude
Tsai et al. [187]	(1) Executive functions (oddball task) during EEG measurements
	(2) Between group comparisons:
	- ↑ P3a amplitude (i.e., at F3 and F4) and P3b amplitude (i.e., at Cz, Pz, and Oz) in RT during CT compared with CON (n)
	- ↑ Accuracy in RT during CT compared with CON (n)
	- ↓ Reaction time in RT during CT compared with CON (n)
	Posttest vs. pretest:
	- ↓ Reaction time in RT during CT
	- ↑ Serum IGF-1 levels in RT
	- ↓ Serum homocysteine levels in RT
	- Higher serum IGF-1 levels in RT were associated with the faster reaction times and larger P3b amplitudes
Tsai et al. [195]	(1) Working memory (Memory span from WAIS-IV); executive functions (Flanker task) during EEG measurements (CT was conducted after exercise cessation when the participant’s body temperature and HR had returned to within 10% of pre-exercise levels, which was on average approximately 5 min after acute resistance exercise cessation.)
	(2) Posttest vs. pretest:
	- ↑ P3 amplitudes (i.e., at Fz, Cz, and Pz, except the Pz electrode in RE) in AE and RE during CT (in all conditions)
	- ↓ Reaction time in AE and RT during CT (congruent and incongruent condition)
	- ↑ Serum IGF-1 in AE and RE; serum BDNF and serum VEGF in AE (prior to cognitive testing at pretest vs. prior to cognitive testing at posttest)
	- ↓ IGF-1 in AE and RE and serum BDNF in AE (prior to cognitive testing at posttest vs. after cognitive testing at posttest)
	- Lower P3 latency across all participants was associated with higher IGF-1 levels (prior to cognitive testing at posttest)
Tsai et al. [191]	(1) Working memory (Memory span from WAIS-IV); executive functions (Task switching) during EEG measurements
	(2) Posttest vs. pretest:
	- ↑ P3 amplitudes in AE and RT
	- ↓ Reaction time in AE and RT during CT (non-switching condition and switching condition)
	- ↑ Accuracy rate in AE and RT during CT (pure condition, non-switching condition, and switching condition)
	- ↑ Serum IGF-1 in RT and serum BDNF in AT
	- ↓ Serum TNF-α and serum IL-15 in RT and AT / ↑ serum TNF-α in CON
	- Higher levels of VO_2max_ are associated with higher levels of serum BDNF in RT and AT
Vonk et al. [183]	(1) Executive functions (Stroop test) during EEG measurements (conducted 10 min, 20 min, 30 min, and 40 min after exercise cessation)
	(2) Posttest vs. pretest:
	- ↓ Response time in RE and LM during CT (congruent and incongruent condition, 10 min after exercise cessation vs. pretest)
	- ↓ Response time in RE and LM during CT (congruent condition, 10 min vs. 30 min after exercise cessation)
	- ↓ Accuracy in RE and LM during CT (incongruent condition, 30 min after exercise cessation vs. pretest)
	- ↑ P3 amplitude in RE and LM during CT (incongruent condition, 30 min and 40 min after exercise cessation vs. pretest)
	- ↑ P3 amplitude in RE and LM during CT (congruent condition, 10 min and 30 min after exercise cessation vs. pretest)
Yerokhin et al. [192]	(1) Cognitive test battery (Stroop test, FOME; CFT); executive functions (oddball paradigm) during EEG
	(2) Posttest vs. pretest:
	- ↓ Beta asymmetry and ↓ N200 A asymmetry
	- ↑ Delta asymmetry
	- ↑ Figure delayed recall and Fuld immediate recall
	- Decreased N200 A asymmetry was significantly correlated with improvements in Fuld immediate and Fuld delayed recall
	- Increase in delta asymmetry was significantly correlated with an improvement in Fuld delayed recall
	(ROI: frontal lobe [FP1, FP2, F7, F8])
Functional and structural magnetic resonance imaging	
Best et al. [184]	(1) Cognitive test battery (Stroop test, TMT A&B, DB, RAVLT, DSST)
	(2) Between group comparisons:
	- ↓ Cortical WM atrophy 2x RT compared with BAT at 2-year follow-up
	- ↑ Executive functions in 1x RT compared with BAT considering changes from baseline to postintervention
	- ↑ Executive functions in 1x RT and 2x RT compared with BAT considering changes from baseline to a 2-year follow-up
	- ↑ Memory performance in 2x RT compared with BAT considering changes from baseline to 2-year follow-up
	- ↑ Peak muscle power in 2x RT compared with BAT considering changes from baseline to postintervention and to a 2-year follow-up
Brinke et al. [197]	(1) Memory (RAVLT)
	(2) Between group comparisons:
	- No significant differences between AT and RT in hippocampal volume after 26 weeks
	- ↑ Hippocampal volume in rt. and lt. hemisphere / total hippocampal volume in AT compared with AT after 26 weeks
	- Positive partial correlation between increase in left hippocampal volume and change in RAVLT (loss after interference condition)
Bolandzadeh et al. [185]	(1) Executive functions (Stroop test)
	(2) Between group comparisons:
	- ↓ Cortical WML volume 2x RT compared with BAT at 2-year follow-up
	- ↓ WML progression in 2x RT at postintervention was associated with maintenance of gait speed
Kjølhede et al. [193]	(1) Working memory & auditory information processing speed (PASAT)
	(2) Changes in cortical thickness in response to RT:
	- ↑ E.g., in subcentral sulcus and gyrus; anterior cingulate sulcus and gyrus, middle anterior cingulate sulcus and gyrus, inferior parietal angular gyrus, inferior temporal gyrus, middle temporal gyrus, temporal pole, superior circular sulcus of insula, superior and transverse occipital sulcus, inferior temporal sulcus, orbital H-shaped sulcus, inferior and superior parts of the precentral sulcus, inferior and superior temporal sulcus
	Between group comparisons regarding cortical thickness:
	- ↑ Anterior cingulate sulcus and gyrus, temporal pole, inferior temporal sulcus, orbital H-shaped sulcus in RT compared with WL after 24 weeks
	- Greater thickness in the temporal pole was correlated with lower EDSS scores
Liu-Ambrose et al. [186]	(1) Cognitive test battery (Stroop test, TMT A&B, DFDB)
	(2) Between group comparisons:
	- ↑ Stroop test performance in 1x RT and 2x RT compared with BAT at 2-year follow-up
	- ↑ Peak muscle power in 2x RT compared with BAT at postintervention and to a 2-year follow-up
	- ↓ Whole brain volume (from baseline) in 1x RT and 2x RT compared with BAT at a 2-year follow-up
	- Improvement in Stroop test performance during intervention was significantly associated with increased gait speed
Liu-Ambrose et al. [45]	(1) Executive functions test (modified Eriksen Flanker task) during fMRI
	(2) Between group comparisons:
	- ↑ Activation of the left anterior insula extending into the lateral orbital frontal cortex in 2x RT compared with BAT at posttest in the incongruent condition
	- ↓ Activation of the left anterior insula extending into the lateral orbital frontal cortex and anterior portion of the left middle temporal gyrus in 2x RT compared with BAT at posttest in the congruent condition
	- ↓ Reduction in interference score (better performance) in 2x RT compared with BAT
Nagamatsu et al. [189]	(1) Cognitive test battery (Stroop test, TMT A&B, DFDB; EPT) and associative memory (memorizing face-scene pairs) during fMRI
	(2) Between group comparisons:
	- ↑ Stroop test performance and associate memory task performance in RT compared with BAT at postintervention
	- ↑ Activation of the right lingual and occipital-fusiform gyri and the right frontal pole in 2x RT during CT compared with BAT at postintervention (encoding and recall of associations)
	- Higher hemodynamic activity in the right lingual gyrus was correlated with better performance in the associative memory test
Suo et al. [190]	(1) Cognitive test battery (e.g. ADAS, TMT A&B, BVRT, COWAT, Category Fluency, SDMT, Logical Memory WMS-III, Matrices WMS-III, Similarities WMS-III)
	(2) Between group comparisons:
	- ↓ ADAS-Cog score (i.e., improved cognition) at posttest in the RT groups compared with all other groups
	- ↑ Posterior cingulate cortex grey matter thickness at postintervention in RT groups compared with all other groups
	- ↓ White matter hyperintensities volumes in the rt. periventricular zone and the rt. parietal zone in RT groups compared with all other groups (significant when analyzed at the regional level / not-significant when whole brain-corrected)
	- Greater posterior cingulate cortex grey matter thickness was significantly correlated with lower ADAS-Cog score (i.e. improved cognition)
	Functional connectivity changes:
	- ↓ PC_FC_ connectivity with the left inferior temporal lobe and the anterior cingulate cortex in RT + SHAM / ↓ PC_FC_ connectivity between the PC and the anterior cingulate cortex in CCT + SHAM
	- ↓ PC_FC_ between the PC and the anterior cingulate cortex in RT + CCT
	- ↑ HIP_FC_ connectivity with the right middle frontal lobe and ↓ connectivity with the right inferior temporal lobe in RT + SHAM
	- ↑ HIP_FC_ connectivity between the hippocampus and the left superior frontal lobe in CCT + SHAM
	- ↑ Hippocampal–anterior cingulate cortex connectivity and the hippocampal–right superior frontal lobe connectivity in RT + CCT
	- ↑ Superior functional connectivity between the hippocampus and the superior frontal lobe is associated with improved memory domain performance

Please note that the sham treatments in Suo et al. [190] were conducted as follows: (i) the cognitive training group (CCT + SHAM) included physical exercises that did not significantly increase heart rate or improve aerobic capacity balance or strength performance (e.g., stretching, toning, and seated calisthenics), and (ii) the resistances exercise group (RE + SHAM) included a computerized, active cognitive control training.

*ADAS-Cog* Alzheimer’s disease assessment scale, *AE* Aerobic exercises, *AT* Aerobic training, *BAT* Balance and toning exercise, *BDNF* Brain-derived neurotrophic factor, *BVRT* Benton visual retention test, *CFT* Complex figure test, *CON (n)* Non-exercising control group, *CON (r)* Control group read magazines, *COWAT* Controlled oral word association test, *CT* Cognitive test, *DB* Verbal digits backward test, *DFDB* Verbal digits forward and verbal digits backward tests, *DSST* Digit symbol substitution test, *EEG* Electroencephalography, *EDSS* Expanded disability status scale, *EPT* Everyday problem solving test, *fMRI* Functional magnetic resonance imaging, *fNIRS* Functional near-infrared spectroscopy, *FOME* Fuld object memory evaluation, *GH* Growth hormone, *HIA* High-intensity aerobic exercise, *HIIT* High-intensity aerobic interval training, *HIRE* High-intensity resistance exercises, *HIRT* High-intensity resistance training, *HOA* Healthy older adults, *IGF-1* Insulin-like growth factor 1, *MCI* Mild cognitive impairments, *MIC* Moderate-intensity exercise combining resistance training and walking, *MCT* Moderate continuous aerobic training, *MIRE* Moderate-intensity resistance exercises, *LM* Loadless movement group, *lt.* Left, *min* Minute, *oxyHb* Oxygenated hemoglobin, *PASAT* Paced auditory serial addition test, *PFC* Prefrontal cortex, *RAVLT* Rey auditory verbal learning test, *RCT* Randomized controlled trials, *RM* Repetition maximum, *RE* Resistance exercises, *RT* Resistance training, *rt.* Right, *SDMT* Symbol digit modalities test, *THI* Total hemoglobin index, *TMT A&B* Trail making test A&B, *TOI* Tissue oxygenation index, *TNF-α* Tumor necrosis factor-alpha, *VEGF* Vascular endothelial growth factor, *VO*_*2max*_ Maximal oxygen uptake during a graded exercise test, *vs.* Versus, *WL* Wait list, *WM* White matter, *WML* White matter lesion volume, *WAIS-IV* Wechsler-IV adult intelligence test, *WMS* Wechsler memory scale, *↑*: significant increase; ↓: significant decrease / F3, F4, F7, F8, FP1, FP2, P3, T3, Cz, Fz, Oz and Pz are specific positions in the international system for EEG electrode placement [199], whereas N1, N2, P1, P2, P3 (P300) constitute specific EEG parameters [200, 201]

## Discussion

### Risk of bias

In general, our results regarding the source of the risk of bias are somewhat heterogeneous (see Fig. 3); nevertheless, the overall quality of the majority of the reviewed studies can be regarded as sufficiently high. However, the risk of bias could be further minimized by proper planning of the study, which would strengthen the plausibility of observed effects. To ensure and enhance the study quality, it appears imperative that future studies report their procedures in sufficient detail (e.g., exercise and training variables) and pay attention to established guidelines such as the CONSORT statement [202] or the STROBE statement [203].

### Selection of participants and study design

The reviewed studies were conducted with healthy young adults, healthy older adults, or older adults with MCI or beginning dementia. Therefore, our knowledge about the effect of resistance exercises and/or resistance training on cognitive functions is limited to these cohorts, and further investigations with other cohorts are required. In particular, older adults with sarcopenia are a key group because there is a high prevalence (ranging from 1 to 33%) of this condition in various older populations [204], which poses major economic costs to the welfare system [205]. Sarcopenia comprises the age-related loss of muscle mass [206–210] but in the literature the term has often been (incorrectly) extended to the age-related loss of muscle function (e.g., muscle strength) [210–219]. The latter one should be referred to as dynapenia which encompasses the age-related loss of muscle function (e.g., loss of muscular strength and power) [209–211, 220]. However, age-related muscular changes (e.g., sarcopenia) could also lead to a decline in cognitive performance [221, 222]. Hence, older adults with sarcopenia and/or dynapenia may profit in two ways (physically and cognitively) from resistance exercises/resistance training.

In the terms of study design, in future resistance exercise and/or resistance training studies, moderator variables such as gender [223–226] or genotype [227, 228], which may influence the effectiveness of the resistance exercise and/or resistance training, should be considered and analyzed. The assessment and analysis of moderators may help provide a better understanding of the observed inter-individual variability regarding the effect of physical exercise (e.g., resistance training) on the brain and on cognitive functions and help to foster the optimization of physical exercise interventions [125]. Furthermore, chronobiological factors (such as circadian variability) should be considered since they affect muscular adaptions in response to resistance exercises [229–232] and affect cognitive performance [233–235]. However, hemodynamic responses are reported to be relatively unaffected by, for instance, circadian variability [236].

Moreover, larger cohorts and longer intervention intervals could be beneficial (especially in [f] MRI studies) for increasing the external validity and for adaptation processes to manifest [237]. In addition, concerning cognitive testing, it seems advisable to use standardized sets of cognitive tests or to employ the latent variable approach (create an unobserved [latent] variable for a distinct set of cognitive tests) [238]. In this context, the ‘human baseline hypothesis’ should be considered, which claims that the baseline values of strength (e.g., grip strength, knee extensor strength) assessed prior to resistance training and/or after a detraining period are a more appropriate indicator of health outcomes than the training-related increase in strength values [239].

With regard to upcoming cross-sectional studies, neuroimaging methods (e.g., fNIRS, see [179]) should be employed as they help to better understand the association between superior cognitive performance (e.g., in global cognitive abilities) and superior muscular performance previously operationalized by (i) hand grip strength [86, 88, 89], (ii) isokinetic quadriceps strength [82, 83], (iii) leg power [84], or (iv) whole-body muscular strength [85].

### Functional brain changes and cognition in response to resistance exercises or resistance training

#### Hemodynamic functional brain changes and cognition

Currently, only a few studies have investigated the influence of resistance exercises and/or resistance training on functional brain parameters in healthy adults during standardized cognitive tasks. However, regardless of whether resistance exercises were conducted as an acute bout [43] or over a period of 16 weeks [44], proxies of cortical activation in the prefrontal cortex during the Stroop test were found to be decreased. In another resistance training study (52 weeks), a decrease in brain activation was observed exclusively during the relatively easy task condition, whereas increased activation was found in the more difficult task condition [45]. These observations stand in contrast to the findings of acute aerobic exercise studies [28, 29, 43] and aerobic training studies [44], in which, in general, increased activation of prefrontal areas during cognitive testing was observed after exercising [180]. Notably, similar to the findings of most aerobic exercise or aerobic training studies, the reviewed resistance exercise and/or resistance training studies also reported improved cognitive functions [43–45]. Hence, decreases in the applied proxies of neuronal activity might indicate more efficient processing or automatization of cognitive processes. Moreover, it is likely that the decrease in brain activation in response to resistance exercises and/or resistance training is related to neurobiological mechanisms different from those induced by aerobic exercises or aerobic training [107, 223, 240]. Future studies are urgently needed to investigate the underlying neurobiological mechanisms of different types of acute physical exercises (e.g., resistance exercises vs. aerobic exercises) and chronic physical training (e.g., resistance training vs. aerobic training). Analysis of the neurobiological changes in response to different physical exercise/training interventions will also contribute to a better understanding of the functional changes in the brain. In this regard, Liu-Ambrose et al. [45] noticed that after the completion of a 52-week long resistance training program, functional brain activations in the left anterior insula extending from the lateral orbital frontal cortex and in the anterior portion of the left middle temporal gyrus during execution of a cognitive task were altered [45]. The left anterior insula, for instance, plays an important role in successful performance in response inhibition tasks [241], which may be based on their involvement in (i) the stopping ability [242], (ii) the assurance of general task accuracy [242], and (iii) maintaining a stable task set control [243, 244]. The left middle temporal gyrus is especially activated in complex Go−/No-Go situations [245]. However, in contrast, in comparable aerobic training, higher task-related activation in prefrontal areas and parietal cortices and decreased activation of the anterior cingulate cortex was observed [246]. Parietal areas [247] and prefrontal areas [248, 249] are involved in a variety of cognitive processes, among them attention [250, 251]. In particular, the parietal areas [252, 253] and the prefrontal areas [254, 255] are strongly involved in selective attention and the frontoparietal network in maintaining and manipulating task-relevant information in working memory [243]. In the context of attentional processes, the anterior cingulate cortex is also an important structure because it allocates attentional resources based on the recruitment of task-appropriate processing centers [256]. Moreover, the anterior cingulate cortex is activated in conflict processing where erroneous responses are highly probable [257–260]. Taken together, resistance training might be beneficial for cognitive processes that aim to avoid unwanted responses (e.g., maintaining stable task set control and increased stop efficacy), whereas aerobic exercises may enhance cognitive processes such as selective attention (e.g., maintaining task-relevant information) [45]. Further research is needed to verify this assumption.

The positive effect of resistance training on brain health is also underpinned by findings of Nagamatsu et al. [189], who observed higher cortical activation during an associative memory task in older individuals with MCI after they had undergone long-term resistance training (52 weeks). Moreover, this higher cortical activity was positively correlated with improvements in cognitive performance [189]. Another mechanism through which resistance training may ensure or/and improve brain health in MCI may be related to the modulation of functional connectivity. It was observed that (i) the resting-state functional connectivity between posterior cingulate cortex and other brain regions is generally decreased in individuals with MCI [261–264], (ii) functional connectivity between the posterior parietal cortex and the temporal cortex is associated with performance on neuropsychological tests [261], and (iii) the resting-state functional connectivity between the hippocampus and other brain regions is disturbed in individuals with MCI [265] or Alzheimer’s disease [266, 267]. Notably, resistance training lasting 26 weeks increases the functional connectivity among the posterior cingulate cortex, the left inferior temporal lobe, and the anterior cingulate cortex and between the hippocampus and the right middle frontal lobe [190]. Based on the mentioned changes in resting-state functional connectivity in neurological diseases (e.g., MCI) and the positive influence of resistance training on resting-state functional connectivity, it can be speculated that resistance training may be a beneficial intervention strategy for ensuring or/and improving brain health and cognition in those cohorts.

#### Neuroelectric functional brain changes and cognition

A higher P3 amplitude (also known as P 300) was observed in younger adults after an acute bout of resistance exercises [182, 183] and in healthy older adults after 48 weeks of resistance training [187]. Furthermore, a higher P3 amplitude was observed in individuals with MCI after an acute bout of resistance exercises [195] or after 16 weeks of resistance training [191]. Elevated P3 amplitudes are generally associated with neural activity and cognitive processes [268, 269]. Upregulation of the P3 amplitude after resistance exercises and/or resistance training may be beneficial for brain health because diminished P3 amplitudes were observed in older individuals [270, 271] and individuals with neurological diseases (e.g., Alzheimer’s disease) [272]. The associations between event-related potentials (e.g., P3 amplitude) and neurotrophic factors obtained after acute resistance exercises [182, 195] and/or resistance training [191] support the “neurotropic hypotheses” [114–117]. Profound changes in neuroelectric outcomes were also observed after 12 weeks of resistance training with decreased resting-state theta power in older adults with and without MCI and increased resting-state alpha power in older adults with MCI [188]. The relevance of these findings is currently unclear because contradictory observations regarding meaningful changes in alpha and theta power are found in the literature. For instance, on the one hand, more resting-state alpha power and less resting-state theta power were associated with better cognitive performance [273, 274], whereas, on the other hand, it has also been reported that higher resting-state theta power is linked to superior cognitive performance (e.g., in category fluency task) [275, 276]. Nevertheless, the notion that resistance training positively affects brain health was clearly confirmed by the observation of statistically significant correlations between neuroelectric changes (e.g., in asymmetry index) and changes in memory performance in older adults in response to a resistance intervention lasting 10 weeks [192]. In addition, Özkaya et al. [194] observed differences in neuroelectric parameters as a function of the type of physical training. This observation supports the idea that resistance and aerobic training have different impacts on the underlying neurobiological processes [223, 225, 240].

In sum, based on the small number of studies, it is too early to draw generalizable conclusions with respect to functional brain changes, but the available results suggest that resistance exercises and/or resistance training can be a promising strategy to ensure brain health. However, further studies are urgently needed to investigate the effect of an acute bout of resistance exercises and/or resistance training on functional brain changes. Here, upcoming studies should also pay attention to the investigation of neurobiological processes that may cause functional brain changes.

### Structural brain changes and cognition in response to resistance training

In response to resistance training over an intervention period of 52 weeks (performed two times per week), (i) a reduction in whole brain volume [186], (ii) a reduction in cortical white matter atrophy [184], and (iii) a reduction in white matter lesions [185] were observed in comparison to training with balance or toning exercises. The reduction in whole brain volume is surprising because, in general, ‘more’ is often associated with ‘better’. However, it is assumed that the reduction in whole brain volume is perhaps caused by the improvement of certain brain pathologies, in particular the removal of amyloid plaques and shifts in cerebral fluids [186, 277, 278], which, in turn, might positively influence brain health. This view is supported by the recent findings of Yoon et al. [279], who observed a relationship between brain amyloid-β levels and hand grip strength (e.g., high levels of brain amyloid-β and low grip strength). The removal of amyloid plaques could be one possible neurobiological mechanism explaining the observed improvements in executive functions [186] because accumulation of amyloid-β plaque is commonly linked to worsened domain-specific cognitive functions (e.g., executive functions and memory) [280–282], and neurological diseases such as Alzheimer’s disease [283–286].

Furthermore, given that white matter abnormalities (e.g., high load of white matter lesions) are linked to a decline in cognitive functions (i.e., global cognition and processing speed) [13, 287–290] and are associated with neurological diseases such as dementia [291, 292], the resistance training-induced changes in white matter (e.g., reduced volume of lesions and reduced atrophy) are likely to be beneficial for brain health. Notably, the reduced volumes of white matter lesions after 52 weeks of resistance training are linked to increased gait speed [185]. Based on the findings that both slower gait speed [293] and white matter lesion load [294] are linked to an increased fall risk, the positive changes within the white matter in response to resistance training suggest that engaging in resistance training could play a substantial role in preservation of the neural correlates of all-day tasks (e.g., safe walking).

In response to resistance training, which was performed twice a week for 26 weeks, grey matter thickness in the posterior cingulate cortex was found to increase significantly [190]. This increase in cortical thickness of the posterior cingulate cortex was linked to improved global cognitive performance [190]. This neurobiobehavioral relationship underpins the assumption that the posterior cingulate cortex is important for cognition, although there is still no agreement about its exact role [295]. However, reductions in metabolism [296] and volume [297] were observed in the posterior cingulate cortex in Alzheimer’s disease. Hence, the possible ability to shape this cortical structure by engaging in resistance training is a promising approach to ensure brain health and to prevent neurological diseases. In the context of neurological diseases, it was also observed that resistance training for 24 weeks increased the cortical thickness in distinct areas, such as the temporal pole, in individuals with MS. The increased cortical thickness in the temporal pole was associated with better scores on the Expanded Disability Status Scale (EDSS), suggesting that resistance training has a positive impact on brain health and functional abilities in this cohort. There are even reports in the literature that a single resistance exercise (leg press) has profound effects on brain volumes (but without a relation to cognitive functions) in healthy older adults. Here, statistically significant increases in grey matter density in the posterior and anterior lobe of the cerebellum, the superior frontal gyrus in the frontal lobe, and the anterior cingulate cortex in the limbic lobe were observed [131]. In summary, these results support the view that robust neuroplastic changes can be evoked through resistance training, which contribute to the maintenance of brain health.

Interestingly, one of the reviewed studies directly compared resistance and aerobic trainings and found no statistically significant difference in hippocampal volume changes between trainings [197]. Although an increase in hippocampal volume was reported after both aerobic [24] and resistance training in older adults [130], few brain imaging studies are currently available that directly compare different types of physical training. For instance, it was observed that dancing conducted for several months led to a greater increase in cortical grey matter in frontal and temporal regions [298–300] and in hippocampal volumes [301] than a combination of resistance, endurance, and flexibility training. Hence, comparing different types of physical interventions (e.g., resistance training vs. aerobic training vs. dancing) with regard to their effectiveness in evoking structural and functional brain changes is an interesting topic for further studies. Such knowledge is necessary to foster the development of individualized physical interventions, which are deemed to be more effective than the ‘one-size-fits-all approach’ [125, 223, 302].

Taken together, resistance training reduces white matter atrophy and increases grey matter volumes in distinct brain areas. Based on the observed relationship between structural changes and behavior [185, 190], the positive role of resistance training in ensuring (and improving) brain health is reinforced. Further studies comparing different types of physical interventions with respect to structural brain changes are required.

### Neurophysiological adaptation processes in connection with resistance exercises and resistance training

Structural brain changes in response to resistance training rely at least partly on the modulation of specific molecular and cellular pathways that are involved in neuroplasticity and – consequently – in positive effects of cognitive performance [112, 240]. In this context, the modulating role of resistance exercises and/or resistance training on the release of neurochemicals such as BDNF, IGF-1, and homocysteine is discussed in the literature [121, 223, 303, 304]. In the following, we briefly outline how these neurochemicals may contribute to the observed functional and structural brain changes.

#### BDNF

In particular, structural brain changes after physical interventions are assumed to be mediated by BDNF [114, 118, 119, 223, 240]. In addition, serum BDNF concentrations have been linked to spatial memory performance [21] and higher serum BDNF concentrations in response to acute physical exercises [305] or physical training [306] have been associated with improvements in executive functions. Furthermore, BDNF is involved in many neuroplastic processes, such as synaptogenesis, long-term potentiation of synaptic transmission, regulation of the differentiation of neuronal precursor cells, and neuronal survival [120]. The important role of BDNF in neuroplasticity is underpinned by the findings that reduced serum BDNF concentrations were linked to a decline in hippocampal volume and that changes in serum BDNF concentrations after aerobic training were associated with hippocampal volume changes [24]. Although hippocampal changes could not be observed in one of the reviewed studies after 26 weeks of resistance training [197], there is solid evidence that resistance exercises (especially at high-load conditions) [307–311] and resistance training (especially in males) [308, 312] upregulate serum BDNF concentrations. Such an increase in response to resistance exercise and resistance training was also reported for plasma BDNF [313]. Notably, it is assumed that concentrations of BDNF stored in immune cells and/or platelets are mirrored in the level of serum BNDF, while plasma BDNF is a marker of the concentration of freely circulating BDNF [314, 315]. Based on the previously mentioned connections between (serum) BDNF, brain physiology, and cognition (i.e., executive functions), it can be speculated that BDNF-driven mechanisms might contribute to neurocognitive changes after resistance exercises and/or resistance training. However, further studies are urgently needed to deepen our knowledge regarding the interrelationship between resistance exercises and/or resistance training-induced expression of (serum) BDNF in humans and its relation to functional and structural brain changes as well as to cognitive performance (as a function of age).

#### IGF-1

Engaging in resistance exercises [316] and resistance training [187, 317] fosters the expression of IGF-1, which is predominantly released by the liver (global output, ~ 70% of total circulating IGF-1), the musculature (local output), and the brain (local output) itself [318, 319]. Because circulating IGF-1 can cross the blood-brain barrier (BBB), locally expressed IGF-1 (e.g., from musculature) is likely to be available in the brain [318, 319]. IGF-1 triggers various mechanisms that contribute to neuroplasticity in the human brain, such as synaptic processes (e.g., long-term potentiation) [320, 321], angiogenesis in the brain, axon outgrowth, dendritic maturation, and synaptogenesis [319, 322]. Moreover, IGF-1 likely plays an important role in structural grey matter changes because it is involved in neuroplastic mechanisms that foster neuronal survival [323] such as (i) proliferation of neural cells [324, 325], (ii) inhibition of apoptosis of neural cells [324, 325], and (iii) protection of neurons against toxicity by, for instance, amyloid peptides [324]. While there is some evidence that higher serum IGF-1 levels are linked to greater total brain volumes [326] or hippocampal volume [327], the exact roles of IGF-1 in the central nervous system remain elusive [328]. However, the assumption that IGF-1-activated pathways play an important role in changing brain function is underpinned by the findings of a reviewed study that reported higher peripheral serum IGF-1 concentrations after 52 weeks of resistance training in healthy older individuals alongside behavioral (e.g., improved accuracy and reaction times in executive function tests) and functional improvements (e.g., P3 amplitude) [187, 191]. Such a relationship between cognitive performance and peripheral serum IGF-1 concentrations would be in accordance with previous findings linking peripheral serum IGF-1 levels to cognitive performance (e.g., global cognition assessed by MMSE) in older individuals [329] and individuals with MCI [330]. Notably, it has also been reported that solely an optimal concentration of peripheral serum IGF-1 is associated with superior global cognition (assessed by MMSE) and processing capacity [331], which could be related to the multiple and divergent roles that IGF-1 plays in the human brain [319, 332]. On the one hand, IGF-1 is linked to beneficial processes (e.g., stimulating synaptogenesis and contributing to neuronal cell survival), but on the other hand, IGF-1 is also associated with detrimental processes (e.g., generation of reactive oxygen species and inhibition of autophagy) [319]. There is currently insufficient evidence to draw firm conclusions regarding the relationship between physical exercise, modulation of IGF-1, structural and functional brain changes, and cognitive functions [333]. Hence, further studies are urgently needed to gain deeper insights into the relationship between exercise-induced modulation of IGF-1 release, functional and structural brain changes, and cognitive performance [332, 333].

#### Homocysteine

A possible neurobiological mechanism that elucidates, at least partly, the effects of resistance training on white matter and cognition could be derived from the known effects of resistance training on the amino acid homocysteine. First, it is important to remember that a higher total homocysteine level is linked to (i) a higher extent of white matter lesions [334], (ii) a higher (faster) brain atrophy rate [335–337], (iii) an increased risk of neurological diseases [338–344], and (iv) poorer global cognitive performance and executive functioning [345–350]. Second, it is known that resistance training decreases the level of plasma [351] and serum homocysteine [187, 352]. Hence, it could be speculated that reducing the homocysteine level in response to resistance training may, at least partly, have positive effects on brain structure (e.g., white matter changes such as reduced atrophy) and/or cognitive functions. However, such relationships have not been directly observed in the studies reviewed [187] and have to be investigated in future studies.

### Influence of exercise variables and training variables on neurocognition

With regard to all studies reviewed, the exercise and training variables of the resistance intervention protocols were chosen as to induce muscle hypertrophy and muscle strength improvements, which is not surprising, as resistance training programs generally focus on improving these two factors. Moreover, this observation is consistent with two other reviews summarizing the results of resistance exercise and resistance training studies on outcomes on a behavioral level [107, 353]. However, given that the dose provided by a physical intervention (e.g., resistance exercise or resistance training) is a function of exercise variables and training variables and that the reviewed studies are relatively homogenous regarding the selection of exercise variables and training variables, our knowledge about the dose-response relationship in resistance exercise and resistance training is relatively meager (especially in view of the fact that resistance exercises and resistance training can be designed in many different ways to focus on different aims for muscular performance). A deeper understanding of the dose-response relationship is needed [105, 108, 110] because the dose (the design of exercise variables and training variables, see Table 3) is a key factor influencing responsiveness [357, 358] and individualizing physical interventions [123, 124, 359].

**Table 3:** Overview of exercise variables and training variables [60, 113, 354–356]

Variables for structuring a single resistance exercise session (exercise variables)	
(i) *Load* (amount of weight that is used for an exercise; usually given as a percentage of the one repetition maximum [1RM])	
(ii) *Number of repetitions*	
(iii) *Number of sets*	
(iv) *Inter-set rest period*	
(v) *Inter-exercise rest period*	
(vi) *Number of exercises* (for the whole training session or for a muscle or a muscle group with the same function)	
(vii) *Repetition velocity* (with respect to the conducted resistance exercise and the starting position, temporal details should be given as follows: i.e., biceps curls starting with fully extended arms [e.g., bench press starting with fully extended arms]: concentric phase [eccentric phase] – inter-repetition rest periods – eccentric phase [concentric phase] – rest period up to the start of the next repetition, e.g., 2–0–2–1 s)	
(viii) *Muscle action* (concentric, eccentric, isometric)	
(ix) *Exercise selection* (e.g., multi-joint or single-joint exercises)	
(x) *Exercise order* (e.g., squat, leg extension, biceps curl, and concentration curl or squat, biceps curl, leg extension, and concentration curl)	
(xi) *Volitional muscle failure*	
(xii) *Range of motion*	
Variables for structuring resistance training (training variables)	
(1.) *Frequency* (number of training sessions per week)	
(2.) *Density* (distribution of training sessions across a week with regard to recovery time in-between training sessions)	
(3.) *Duration* (duration over which a training program is carried out; e.g., before exercise variables will be changed)	

Please note, that some exercise variables are usually summarized into variables with different designations: e.g., *volume* [exercise variables (ii), (iii), and (iv)], *time under tension* [TUT, sum of the exercise variables (ii) and (vii)] or *duration of an exercise session* [depends on exercise variables (ii), (iii), (iv), (v), (vi), (vii), and the duration of warm-up and cool-down] [354, 356]

In the following section, we outline promising starting points for investigating the dose-response relationship in resistance exercise and/or resistance training studies.

With regard to *load*, on the behavioral level, it was observed that an acute bout of moderate-load resistance exercises (70 to 100% of the 10RM, 10RM = the load needed for 10 repetitions until maximum exhaustion) improves the speed of processing, while resistance exercises with low load (40% of the 10RM) improve executive functions [138]. Furthermore, it was reported that improvements in executive functions were larger after moderate-load (70% of 10RM) than low-load (40% of 10RM) resistance exercises [156]. The finding that resistance exercises with moderate loads are especially beneficial for cognitive performance is supported by the observation that resistance exercises with moderate loads (60% 1RM) lead to larger positive effects on higher cognitive functions (i.e., Stroop interference score) compared with resistance exercises with heavier loads (≥ 75% 1RM) [360]. In another study, it was noticed that a single bout of high-load (100% of 10RM) resistance exercises resulted in less interference and fastened reaction times for the Stroop task 15 min after exercise cessation, while 180 min after exercise cessation, low-load (40% of 10RM) and moderate-load (70% of 10RM) resistance exercises were associated with increased performance on the plus-minus and the Simon task [146]. However, at the moment, only two studies have employed neuroimaging methods to investigate the dose-response relationship with respect to the exercise load [182, 183]. In this study, no statistically significant differences in neuroelectric outcomes between conditions were observed [182, 183]. Based on the sparse evidence in this area, further research is required to investigate whether such load-dependent cognitive improvements are mirrored in acute processes of the central nervous system (e.g., measured prior and after resistance exercises by fNIRS [180] or EEG [201, 360–362].

With regard to *number of sets*, on the behavioral level, it was reported that younger adults performing three or five sets of a resistance exercise showed after a 8-week intervention period greater improvements in inhibitory control (i.e., assessed by accuracy and mean response time in the Stroop test) than younger adults performing one set of the same resistance exercise [363]. Because the above-mentioned study did not apply neuroimaging techniques or quantify neurotrophic markers (e.g., BDNF) [363], future investigations are needed to elucidate the underlying neurobiological mechanisms.

With regard to *frequency*, on the behavioral level, resistance training three times a week was more efficient than training twice a week [109]. Since most reviewed studies conducted resistance training twice a week [45, 184–186, 189, 190] and observed beneficial results or did not compare a training with two sessions per week to other training frequencies [44, 187], the findings of Li et al. [109] are not supported by functional or structural data. Hence, future studies are required to investigate the influence of training frequency on functional and structural brain changes (e.g., one time per week vs. three times per week).

Since changes at the molecular and cellular levels (e.g., metabolic response, such as peripheral blood lactate concentration) are linked to behavioral changes, a promising approach to positively influence neurocognition could be the alteration of molecular and cellular processes by adjusting the exercise prescription via exercise and training variables.

In particular, after an acute bout of physical exercise, postexercise concentrations of peripheral blood lactate were found to be linked to improvements in executive functions [364–366]. In this context, peripherally (e.g., in the musculature) released lactate is expected to be utilized as ‘fuel’ for cognitive processes because it can cross the BBB with the help of monocarboxylate transporters [367–371]. Furthermore, peripheral lactate may trigger the release of serum BDNF [309, 311, 372], but this relationship seems to be highly reliant on the correct selection of resistance exercise variables [309]. Notwithstanding, it has been well demonstrated that serum BDNF contributes significantly to changes in brain structure [21, 24] and performance (e.g., cognition) [21, 305, 306]. Consequently, given that the peripheral concentration of blood lactate is a function of resistance exercise variables such as repetition velocity [373, 374] or inter-set rest periods [375], it seems reasonable to speculate that a purposeful modification of these exercise variables may also influence neurocognition outcomes. Notably, in this context, it was also hypothesized that resistance exercises with blood flow restriction (BFR) could be beneficial for neurocognition because resistance exercises with BFR or resistance training with BFR induce beneficial processes on a molecular and cellular level (for review see [113]). However, further research in this area with a strong focus on underlying neurobiological processes, functional and structural brain changes, and cognition is required.

Finally, similar to the major ongoing discussions regarding which variables may be optimal to improve muscular adaptions, such as muscle hypertrophy or strength [376–390], the optimal exercise prescription (e.g., exercise variables and training variables) for resistance exercises and/or resistance training with respect to brain health (including appropriate functional and structural brain changes as well as enhancement of cognitive functions) are largely unknown and have to be elucidated in future studies [105, 108, 110]. In addition, the interested reader may find further and more detailed information regarding the design of resistance exercise sessions or resistance training in the referenced literature [355, 391–394].

## Recommendations for future studies

- Based on the available evidence derived from the reviewed studies and other recommendations [107], resistance exercises and/or resistance training aiming to enhance cognitive functions and evoke positive functional and structural brain changes should be designed to induce muscle hypertrophy.
- Future studies are needed to investigate the influence of the adjustment of different resistance exercise variables (e.g., load, number of sets, training frequency, training duration) on functional and structural brain changes in conjunction with cognitive functions.
- To understand the time-course of functional and structural brain changes, neuroimaging should be performed at several time points after an acute bout of resistance exercise or during the resistance training intervention.
- The inclusion of further cohorts (e.g., older individuals with sarcopenia and/or dynapenia) is needed to verify whether resistance exercise-induced improvements also occur in such needy cohorts and how this is related to functional and structural brain changes.
- Interventional studies (or cross-sectional studies) investigating the relationship of resistance exercises (or strength, muscle function/structure) and cognition should utilize different neuroimaging methods during standardized cognitive testing and assess neurochemical substances (e.g., neurotransmitters, neurotrophic factors) to elucidate underlying neurobiological mechanisms.
- Bed rest studies, which reported a worsening of executive functions [395–397], profound brain changes [397–399], and a decrease in muscle mass as well as muscle strength [400–408], could be an interesting model to study the relationship between the muscular system, functional and structural brain changes, and cognition.

## Conclusions

In summary, resistance exercises and resistance training are powerful physical intervention strategies to induce meaningful functional brain changes, especially in the frontal lobe, which are accompanied by improvements in executive functions. Furthermore, based on the studies reviewed, resistance training leads to lower white matter atrophy and lower volumes of white matter lesions. However, given the small number of available studies that have mostly been part of greater study projects (Brain Power Study and SMART [Study of Mental and Resistance Training]), further research investigating the influence of an acute bout of resistance exercise and chronic resistance training on cognition and the underlying neurobiological mechanisms (e.g., functional and/or structural brain changes) is needed. This future research should also focus on the effects of systematically manipulating exercise and training variables (dose-response relationship) and further including specific cohorts with the greatest need (e.g., older individuals with sarcopenia and/or dynapenia). Most importantly, engaging regularly in resistance exercises and/or resistance training across the whole lifespan appears to be imperative for ensuring physical and brain health because muscular weakness in the early years of life (e.g., adolescence) has been shown to be associated with disability in later life (e.g., after 30 years) [409] and even 4 weeks of detraining (being physical inactive) completely reversed the physical and cognitive improvements of 22-week resistance training in older adults [410]. Hence, to summarize in a metaphorical sense: “May the force be with you across your lifespan.”

## Data Availability

Not applicable.
